# Cathepsin F Cysteine Protease of the Human Liver Fluke, *Opisthorchis viverrini*

**DOI:** 10.1371/journal.pntd.0000398

**Published:** 2009-03-24

**Authors:** Porntip Pinlaor, Natthawut Kaewpitoon, Thewarach Laha, Banchob Sripa, Sasithorn Kaewkes, Maria E. Morales, Victoria H. Mann, Sandi K. Parriott, Sutas Suttiprapa, Mark W. Robinson, Joyce To, John P. Dalton, Alex Loukas, Paul J. Brindley

**Affiliations:** 1 Department of Pathology, Khon Kaen University, Khon Kaen, Thailand; 2 Faculty of Allied Medical Sciences, Khon Kaen University, Khon Kaen, Thailand; 3 Department of Tropical Medicine, Tulane University Health Sciences Center, New Orleans, Louisiana, United States of America; 4 Department of Parasitology, Khon Kaen University, Khon Kaen, Thailand; 5 Department of Microbiology, Immunology & Tropical Medicine, George Washington University Medical Center, Washington, D.C., United States of America; 6 Institute for the Biotechnology of Infectious Diseases, University of Technology Sydney, Sydney, New South Wales, Australia; 7 Division of Infectious Diseases, Queensland Institute of Medical Research, Brisbane, Queensland, Australia; Moredun Research Institute, United Kingdom

## Abstract

**Background:**

The liver fluke *Opisthorchis viverrini* is classified as a class I carcinogen due to the association between cholangiocarcinoma and chronic *O. viverrini* infection. During its feeding activity within the bile duct, the parasite secretes several cathepsin F cysteine proteases that may induce or contribute to the pathologies associated with hepatobiliary abnormalities.

**Methodology/Principal Findings:**

Here, we describe the cDNA, gene organization, phylogenetic relationships, immunolocalization, and functional characterization of the cathepsin F cysteine protease gene, here termed *Ov-cf-1*, from *O. viverrini*. The full length mRNA of 1020 nucleotides (nt) encoded a 326 amino acid zymogen consisting of a predicted signal peptide (18 amino acids, aa), prosegment (95 aa), and mature protease (213 aa). BLAST analysis using the *Ov*-CF-1 protein as the query revealed that the protease shared identity with cathepsin F-like cysteine proteases of other trematodes, including *Clonorchis sinensis* (81%), *Paragonimus westermani* (58%), *Schistosoma mansoni* and *S. japonicum* (52%), and with vertebrate cathepsin F (51%). Transcripts encoding the protease were detected in all developmental stages that parasitize the mammalian host. The *Ov-cf-1* gene, of ∼3 kb in length, included seven exons interrupted by six introns; the exons ranged from 69 to 267 bp in length, the introns from 43 to 1,060 bp. The six intron/exon boundaries of *Ov-cf-1* were conserved with intron/exon boundaries in the human cathepsin F gene, although the gene structure of human cathepsin F is more complex. Unlike *Ov*-CF-1, human cathepsin F zymogen includes a cystatin domain in the prosegment region. Phylogenetic analysis revealed that the fluke, human, and other cathepsin Fs branched together in a clade discrete from the cathepsin L cysteine proteases. A recombinant *Ov*-CF-1 zymogen that displayed low-level activity was expressed in the yeast *Pichia pastoris*. Although the recombinant protease did not autocatalytically process and activate to a mature enzyme, *trans*-processing by *Fasciola hepatica* cathepsin L cleaved the prosegment of *Ov*-CF-1, releasing a mature cathepsin F with activity against the peptide Z-Phe-Arg-NHMec >50 times that of the zymogen. Immunocytochemistry using antibodies raised against the recombinant enzyme showed that *Ov*-CF-1 is expressed in the gut of the mature hermaphroditic fluke and also in the reproductive structures, including vitelline glands, egg, and testis. *Ov*-CF-1 was detected in bile duct epithelial cells surrounding the flukes several weeks after infection of hamsters with *O. viverrini* and, in addition, had accumulated in the secondary (small) bile ducts where flukes cannot reach due to their large size.

**Conclusions/Significance:**

A cathepsin F cysteine protease of the human liver fluke *O. viverrini* has been characterized at the gene and protein level. Secretion of this protease may contribute to the hepatobiliary abnormalities, including cholangiocarcinogenesis, observed in individuals infected with this parasite.

## Author Summary

Opisthorchiasis, oriental liver fluke infection, is a food-borne parasitic disease that afflicts millions of residents in northern Thailand and Laos. Related infections occur in North Asia, including China and Korea. This kind of liver fluke infection is the consequence of eating certain uncooked or undercooked freshwater fish contaminated with the larvae of the parasite *Opisthorchis viverrini*. Whereas the infection can cause disease in the bile ducts and liver, infection with the oriental fluke can lead to the development of a liver cancer, cholangiocarcinoma (bile duct cancer). Our recent studies have begun to focus on products and metabolites from the parasite that are carcinogenic. Many proteolytic enzymes are known to be secreted by parasites. This report centers on a specific category of protease, termed cathepsin F. We determined here that *O. viverrini* expresses a cathepsin F in its gut and in other organs. In the liver fluke, cathepsin F likely plays a role in digesting ingested human cells. The gene encoding the parasite enzyme shows evolutionary relatedness to a similar gene in humans. The fluke cathepsin F also is released from the parasite into livers of infected mammals, where it appears to contribute to inflammation surrounding the parasite. In this regard, it may be involved in early events that lead to bile duct cancer.

## Introduction

*Opisthorchis viverrini* is an important human food-borne pathogen endemic in mainland Southeast Asia, predominantly Northeast Thailand [1],[2]. Infection with this liver fluke parasite causes opisthorchiasis, which is associated with a number of hepatobiliary abnormalities, including cholangitis, obstructive jaundice, hepatomegaly, cholecystitis, cholelithiasis and cholangiocarcinoma. *O. viverrini* infection induces pathological changes including epithelial desquamation, epithelial and adenomatous hyperplasia, goblet cell metaplasia, inflammation, periductal fibrosis and granuloma formation [3]. Experimental and epidemiological findings implicate *O. viverrini* infection in the etiology of cholangiocarcinoma (CCA), cancer of the bile ducts (reviewed in [1]). *O. viverrini* is one of only two metazoan pathogens of humans that is considered a Group 1 carcinogen [4],[5].

A number of studies suggest that inflammation of the bile ducts caused by *O. viverrini* infection and induction of endogenous nitric oxide are important factors for cholangiocarcinogenesis [6],[7]. Other studies have related cell proliferation induced by *O. viverrini*, its antigens and metabolites, and exposure to exogenous carcinogens such as nitrates, nitrites and even *N*-nitroso compounds found in fermented or preserved foods such as *pla-ra* (traditional Thai fermented fish), as factors involved with parasite-associated cholangiocarcinogenesis [8]. The pathogenesis of *O. viverrini*-mediated hepatobiliary changes, which in turn can lead to CCA, can be attributed to greater or lesser degree to mechanical irritation caused by the liver fluke suckers and to the action of molecules excreted or secreted by the parasite [1].

There may be informative analogies to be drawn between *O. viverrini*-induced CCA and the development of stomach cancer caused by infection with CagA-positive strains of *Helicobacter pylori*. In the latter situation, the CagA antigen is delivered by the *H. pylori* bacterium into gastric epithelial cells, where it undergoes tyrosine phosphorylation. Phosphorylated CagA activates SHP-2 tyrosine phosphatase, causing morphological transformation of the infected cell to the hummingbird phenotype. CagA also destabilizes the E-cadherin/beta-catenin complex to elicit aberrant activation of the beta-catenin signal. These events in signal dysregulation underlie stomach cell metaplasia [9],[10]. Whereas our understanding of cholangiocarcinogenesis is less advanced than with *H. pylori*-associated gastric adenocarcinoma, CCA—in like fashion to gastric adenocarcinoma—is an epithelial cell adenocarcinoma associated with a gastrointestinal tract pathogen.

In like fashion to CagA-associated stomach cancer, it is conceivable that products secreted or released by *O. viverrini* into the neighboring bile duct epithelia might promote cholangiocarcinogenesis. The parasite-released mediators might down-regulate apoptosis and/or they may stimulate epithelial cell growth. Accordingly, we have begun to examine the secretome of *O. viverrini* proteome with a particular interest in proteolytic enzymes since they are prominent components of ES of helminth parasites at large [11]–[13]. Recently we reported the biochemical characterization of cysteine protease activities in extracts of several developmental stages of *O. viverrini* [14]. We demonstrated that *O. viverrini* expresses clan CA-like cysteine protease activity with elevated expression in the metacercariae, suggesting that this enzyme activity might participate in larval excystation during mammalian infection. The proteolytic activity was also detected in excretory/secretory (ES) products of sexually mature parasites [14].

In the present report, we have identified a transcript and its genome locus encoding a cathepsin F-like cysteine protease from *O. viverrini*, which we termed *Ov*-CF-1. Analysis of the genomic structure of the *Ov-cf-1* gene revealed conserved exon/intron boundaries with the gene encoding human cathepsin F, although the human gene exhibits a more complex structure including a zymogen with a cystatin domain found within the pro-segment that is absent from the *O. viverrini* gene. Phylogenetic analysis revealed that the deduced *Ov*-CF-1 protease was an orthologue of cathepsin F cysteine proteases, a clade distinct from the cathepsin L proteases described in several related liver and blood flukes. Nevertheless, characterization of a functionally active recombinant form of *Ov*-CF-1 shows that this enzyme cleaves diagnostic peptides (Z-Leu-Arg-NHMec, Z-Phe-Arg-NMHec) that are also cleaved by cathepsin L. *Ov*-CF-1 is secreted by adult *O. viverrini* and immunocytochemical studies localized the protease in the cecum of the adult stage of the parasite. Liberation of *Ov*-CF-1 protease by adult parasites residing in the bile duct may contribute to the pathologies associated with *O. viverrini*-induced hepatobiliary abnormalities.

## Materials and Methods

### *Opisthorchis viverrini*; Genomic DNA and RNA Extraction; RT-PCR

Metacercariae of *O. viverrini* were obtained by digestion with pepsin of flesh of naturally infected cyprinoid fish collected from an endemic area of Khon Kaen province, Thailand. About 100 metacercariae of *O. viverrini* were used to infect hamsters, *Mesocricetus auratus*, by stomach intubation, as described [15]. Infection of hamsters with liver flukes and maintenance of hamsters was carried out at the animal facility of the Faculty of Medicine, Khon Kaen University, using procedures approved by the Animal Ethics Committee of Khon Kaen University. The hamsters were euthanized six weeks after infection, after which the adult *O. viverrini* flukes were perfused from the bile ducts with phosphate-buffered saline (PBS), pH 7.2. The worms were washed with sterile saline, after which genomic DNA (gDNA) was extracted using kits from Gentra Systems (Minneapolis, MN) for long range PCR or Bio-Rad (Hercules, CA) for inverted PCR templates. Eggs of *O. viverrini* were recovered from tissue culture medium where they had been discharged from adult worms [16]. Total RNA was isolated from adults, eggs and metacercariae of *O. viverrini* using Trizol [16],[17]. Contaminating gDNA was removed by treatment of RNA with DNase I (Promega, Madison, WI). For reverse transcription-PCR (RT-PCR), first-strand cDNA was produced with an oligo (dT) primer from 1.0 µg of total RNA using SUPERSCRIPT III reverse transcriptase (Invitrogen, Carlsbad, CA) at 42°C for 60 min. One µl of the resultant cDNA was employed as template in the presence of primers specific for *Ov-cf-1* (5′-TCGGACCAGTATTGGACCAAG -3′ and 5′-TACGCTGGAAAGCACACAACG), with thermal cycling conditions of 30 sec denaturation at 94°C, 30 sec annealing at 55°C and 30 sec extension at 72°C for 30 cycles. Control RT-PCR reactions were performed without reverse transcriptase to ensure that amplified products were derived from cDNA and not contaminating genomic DNA. PCR products were subjected to electrophoresis through 1% agarose and visualized under UV light after staining with ethidium bromide.

### Homology and RACE-PCR Approaches

**Figure 1:**
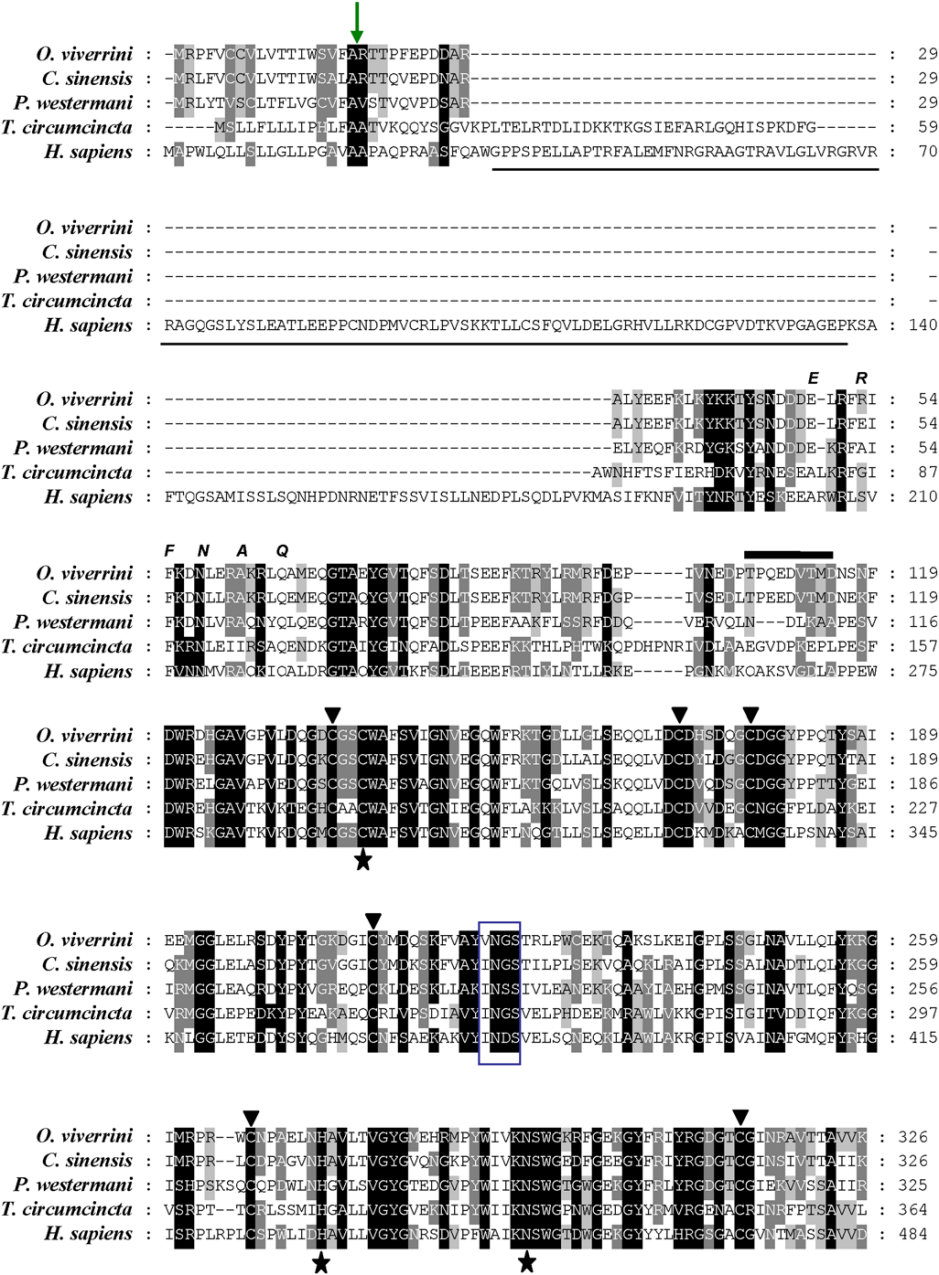
Multiple sequence alignment of deduced amino acids of the cathepsin F from *Opisthorchis viverrini Ov*-CF-1 (AAV69023). The alignment includes other members of the Clan CA, family C1 peptidase family, including CsCF-6 from *Clonorchis sinensis* (ABK918111) and orthologues from *Paragonimus westermani* (AAY81944), *Teladorsagia circumcincta* (ABA01328) and *Homo sapiens* (NP_003784). (The *T. circumcincta* orthologue was included as a representative of a nematode helminth sequence, while human cathepsin F sequence was included to highlight the large prosegment with a cystatin domain, underlined, that exists in some cathepsins F.) Identical residues in more than 50% of sequences are presented in black boxes. Conserved substitutions are identified by grey boxes. The conserved active site cysteine, asparagine and histidine residues are highlighted (stars). The protease-susceptible region at the junction of the prosegment and mature domain is indicated by the thick black line. This region contains the cleavage sites for release of the prosegment from the mature domain and includes the FhCL1 cleavage site for *Ov*-CF-1, VT↓MDNSNFD, and the cleavage site in human cathepsin F, DL↓APPEWD, determined by X-ray crystallography [50]. The thin (green) arrow indicates the position of signal peptide cleavage site. The arrow heads indicate six cysteine residues likely involved in disulfide bridges. The box indicates a conserved, putative *N*-linked glycosylation site, NGS in the mature enzyme domain. The position of the ERFNAQ propeptide motif, conserved in cathepsin F, is indicated.

To search for transcripts encoding Clan CA, Family C1 cysteine proteases of *O. viverrini*, degenerate primers were designed to target conserved domains of papain-like cysteine proteases. The degenerate forward primer, 5′-TGYGGNTCNTGYTGGGCDTTYTCN targeted the conserved CGSCWAFV residues and the reverse primer, 5′-CCARCTRTTYTTBACDATCCARTA targeted the conserved YWIVKNSW residues (Figure 1), where B = C/G/T; D = A/G/T; N = A/C/G/T; R = A/G; Y = C/T. These primers were employed to amplify target sequences from cDNA of adult *O. viverrini*. In addition, specific primers *Ov-cf-spf1* (5′-TGTGGTTCGTGTTGGGCATTTTCT) and *Ov-cf-spr1* (5′-CCAACTGTTTTTGACCGTCCAGTA) targeting the same regions were designed based on the nucleotide sequence of *Clonorchis sinensis* cathepsin F (DQ909018). PCR was performed as follows; 95°C for 5 min followed by 35 cycles of 94°C for 30 sec, 55°C for 30 sec, 72°C for 30 sec and finally, 72°C for 7 min. Fragments of cDNA sequence encoding an *O. viverrini* cysteine protease were obtained by using rapid PCR amplification of cDNA ends (RACE); 5′- and 3′-RACE were performed using specific primers *Ov-cf-spf1* and *Ov-cf-spr1* (above) paired with linker specific primers (Invitrogen). Subsequently, a full length transcript termed *Ov-cf-1* was obtained by PCR using primers designed from nucleotide sequences obtained from 5′- and 3′-RACE products.

### Inverse and Long Range PCR Approaches

Flanking and 5′-UTR sequences upstream of the *Ov-cf-1* gene were amplified using inverse PCR (iPCR) [18]. *O. viverrini* genomic DNA (gDNA) was digested with *Hin*d III, after which the fragments (50 ng/µl) were circularized using T4 DNA ligase (New England Biolabs, Ipswich, MA). One hundred ng of circularized gDNA fragments was employed as template for amplification of the putative 5′-UTR of the gene. Primers IPCRCPFw (5′-CAGGCTCGAATGGGGTAGTTCTGGC) and IPCRCPRe (5′-GCCCGGGCACTATACGAGGAGTTCA) were derived orientated in reverse direction to the mRNA of *Ov-cf-1* (AY821800). iPCR reactions were carried out in 100 µl in 50 mM KCl, 10 mM Tris-HCl (pH 9.0 at 25° C), 0.1% Triton X-100, 1.5 mM MgCl_2_, 0.5 mM dNTP, 0.5 U *Pfu*TurboCx Hotstart DNA polymerase (Promega), with thermal cycling conditions of 94° C for 2 min, followed by 30 cycles of 94° C for 60 sec, 58° C for 60 sec and 72°C for 2 min, and a final extension at 72°C for 10 min. Products were cloned into plasmid TOPO XL PCR (Invitrogen) and nucleotide sequence of inserts determined. In addition, toobtain a genomic fragment spanning the entire gene locus, the *Ov-cf-1* gene locus was amplified from gDNA of *O. viverrini* with the primers specific for the 5′- (CPFw, 5′-GACCTTTCGTGTGTTGCGTGTT) and 3′-termini (CPRe, 5′-GGGCAAAATATCAACATGGAG) of the transcript (GenBank AY821800) using LongRange PCR DNA polymerase (Qiagen, Tubingen, Germany). These amplifications were performed in 50 µl reaction volumes using 100 ng gDNA, 1× LongRange PCR buffer, 1× Q-solution, 0.4 µM of each primer, 2.5 mM MgCl_2_, 500 µM of each dNTP, 2 units of LongRange PCR DNA polymerase. After an initial denaturation step at 93°C for 3 min, 40 thermal cycles of 93°C for 15 sec, 50°C for 30 sec, 68°C for 15 min were carried out, followed b a final step of 68°C for 7 min. PCR products were purified (Gel DNA extraction kit, Real Genomics, Real Biotech Corporation, Taipei, Taiwan) and ligated to the vector pCR4-TOPO (Invitrogen). The ligation products were used to transform *E. coli* competent cells (One shot Mach1-T1; Invitrogen) which were cultured in the presence of ampicillin. Plasmid DNA was isolated from clones, and the sequence of long range PCR products determined by gene walking with gene specific primers.

### Bioinformatics

Bioinformatics analyses to predict potential open reading frames (ORFs), signal peptides, and/or transmembrane domains, conserved domains, and molecular mass were undertaken using ExPASy (http://expasy.org), including the Compute pI/MW tool, Signal P (www.cbs.dtu.dk/services/SignalP), and TMPred 3.0 (www.ch.embrut.org/software/TMPRED_form.html). Assembly of contiguous sequences and multiple alignments were performed with BioEdit software version 7.0.0, http://www.mbio.ncsu.edu/BioEdit/bioedit.html [19]. Analysis of gene loci using BLAST searches at NCBI was undertaken to determine exon and intron structures and boundaries of the *O. viverrini* cysteine protease gene, upstream regulatory sequences, and identities of unrelated sequences populating the introns. Positions of splice sites were predicted using NetGene2 program, http://www.cbs.dtu.dk/services/NetGene2/ [20]. For phylogenetic analysis, the amino acid sequence of *Ov-cf-1* (GenBank AY821800) was aligned with cathepsin F-like and cathepsin L-like, and related cysteine proteases of a variety of eukaryotes using ClustalW algorithms in the BioEdit software suite [21]. Species names and accession numbers of contributing sequences are provided in the phylogram (below). Cathepsin B from *Schistosoma japonicum* (AY222871) was used to root the tree. Multiple sequence alignments were calculated with the distance matrix method using Prodist and Neighbor in Phylip software packages to construct neighbor joining trees. Trees were analyzed under 1,000 bootstrap resampling values, and drawn with TreeView [22].

### Expression and Purification of Recombinant *Ov*-CF-1

The sequence encoding the proform of *Ov*-CF-1 was amplified from cDNA of adult *O. viverrini* using the primers (*Ov*-*cp*-f1) 5′-CGCGCGGAATTCAGAACTACCCATTCGAG and the (*Ov*-*cp*-r1) 5′-CGCGCGTCTAGACGTTTGACAAGGCTGTAGT, which include restriction sites for *Eco*R I and *Xba* I (underlined). PCR products were cloned into the yeast expression vector pPICZα (Invitrogen) using *Eco*R I and *Xba* I restriction sites, and the reading frame confirmed by sequencing. The recombinant plasmid was linearized with *Pme* I and employed to transform the ×33 strain of *Pichia pastoris* (Invitrogen) by electroporation. Transformed yeasts were selected on 1 mg/ml zeocin (Invitrogen) Yeast Peptone Dextrose (YPD) plates, screened by PCR using *Ov-cf-1* gene specific primers, and positive colonies investigated for protein expression by immunoblotting using a monoclonal antibody specific for the hexa-His tag (Invitrogen). A 1.5 litre *P. pastoris* culture was fermented using a clone that exhibited strong expression. The culture supernatant at 96 hours after induction was concentrated to 200 ml by ultrafiltration through a 10 kDa cut off membrane (Pall Scientific), and buffer exchanged into 50 mM NaH_2_PO_4_, 300 mM NaCl, 10 mM imidazole (binding buffer). Recombinant enzyme was affinity purified on Ni-NTA resin (Qiagen), buffer exchanged into PBS, and protein concentration determined using the bicinchoninic acid assay (Pierce, Rockford, IL).

### Antiserum to Fluke Enzyme, Immunoblots, Immunolocalization

Antiserum against affinity purified *Ov*-CF-1 was produced by immunization of a male New Zealand White rabbit. Pre-immune blood was collected from the marginal ear vein two days prior to the first injection. For the first immunization, the rabbit was injected subcutaneously with 1.0 mg of recombinant *Ov*-CF-1 emulsified with an equal volume of Freund's complete adjuvant. The second and third immunizations were carried out with 1.0 mg recombinant protein formulated with Freund's incomplete adjuvant. Immunizations were conducted on days 1, 15 and 29, blood was collected two weeks after the third immunization, and its serum was separated. The specific anti-*Ov*-CF-1 IgG titer of the rabbit serum was determined by enzyme linked immunosorbent assay, and antiserum was stored at −20°C until needed. Cysteine protease activities were enriched using thiol-sepharose chromatography from ES products and preparations of soluble adult *O. viverrini* worms (somatic preparation), as detailed [14]. ES and somatic fractions eluted from the thiol-sepharose were sized by SDS-PAGE, electro-transferred to nitrocellulose, and probed with the rabbit antiserum in order to determine the presence of the *Ov*-CF-1.

*O. viverrini* adult worms or liver tissue from hamsters infected with *O. viverrini* (weeks 1–24) were fixed and cut with a microtome into sections of 4 µm [23]. The sections were deparaffinized in xylene, hydrated in a series of ethanol and water, respectively. Endogenous peroxidase was eliminated by incubation of the sectioned tissues in 5% H_2_O_2_ in methanol for 30 min. Subsequently, sections were washed in water and PBS, and non-specific staining was blocked by incubation in 5% pre-immunization rabbit serum in PBS for 30 min. Sections were probed with rabbit anti-*Ov*-CF-1 antiserum or pre-immunization serum from the same rabbit diluted at 1∶100 (v/v) in PBS at 4°C overnight. After rinsing 3×5 min with PBS, sections were incubated with HRP-conjugated goat anti-rabbit IgG for 1 h. Sections were rinsed with PBS, 2×10 min, after which bound antibody was detected using the substrate diaminobenzidine (DAB). Sections were counterstained with Mayer's hematoxylin, dehydrated, cleared in xylene and mounted in Permount. Images of sections were recorded using a digital camera (Nikon DXA 1200 C) fitted to an Olympus model BX40 compound microscope.

### Biochemical Analysis, Activation, and Processing of Recombinant *Ov*-CF-1

To determine whether the recombinant *Ov*-CF-1 was capable of auto-catalytic activation, 20 µl enzyme (100 µg) was added to 100 µl activation buffer (0.1 M sodium acetate, pH 4.5, 1 mM DTT, 1.25 mM EDTA) and incubated at 37°C. Aliquots of 10 µl were removed at various time points, transferred into tubes containing 1 µl of 1 mM E-64 to halt the enzymatic reaction. The reaction products were analyzed by 4–12% Bis-Tris NuPage gel (Invitrogen) electrophoresis. *Trans*-processing of recombinant *Ov*-CF-1 was undertaken by mixing 50 µg purified recombinant enzyme with 5.0 µg of recombinant *O. viverrini* asparaginyl endopeptidase [17] or an activated recombinant *Fasciola hepatica* cathepsin L (FhCL1) [24] in 0.1 M sodium acetate, pH 4.5, 1 mM DTT , 1.25 mM EDTA in a total reaction volume of 150 µl. The mixture was incubated for 180 min at 37°C, and samples (10 µl) were removed at intervals for analysis (as above).

N-terminal sequencing was performed on recombinant *Ov*-CF-1 and samples taken at time 180 min following *trans*-processing with FhCL1. Following 4–12% Bis-Tris NuPage, proteins were transferred to a polyvinylidene fluoride immobilon-P membrane (Millipore) at 120 mA for 45 min. The membrane was washed with distilled water and stained with 0.025% Coomassie Brilliant Blue R-250 in 40% methanol, 10% acetic acid. Selected protein bands were subjected to 5 cycles of N-terminal (Edman) sequencing at the Biomolecular Research Facility (BRF) at the University of Newcastle (NSW, Australia). To analyze the enzyme specificity of *Ov*-CF-1 and to monitor the course of activation during the *trans*-processing experiments, we employed three fluorogenic peptide substrates with different residues at the P2 position, Z-Phe-Arg-NHMec, Z-Leu-Arg-NHMec and Z-Pro-Arg-NHMec (Bachem). Assays were performed in 0.1 M sodium acetate, pH 4.5, 1 mM DTT, 1.25 mM EDTA (200 µl) in 96-well plates. Reaction kinetics were monitored over 150 min at 37°C by measuring the release of the fluorogenic leaving group (NHMec) at 370 nm excitation, 460 nm emission wavelength, using a Bio-Tek KC4 microfluorometer. Reactions with each fluorogenic substrate included (a) no enzyme control, (b) recombinant *Ov*-CF-1 alone, (c) recombinant FhCL1 alone, and (d) *Ov*-CF-1 +FhCL1.

## Results

### A Transcript Encoding a Cathepsin F-like Protease from Adult *O. viverrini* Liver Fluke

We undertook PCR investigations targeting conserved active site encoding regions of clan CA peptidases employing (a) degenerate primers or (b) primers specific for the *C. sinensis* cathepsin F gene (DQ909018). The former approach did not amplify products of the expected size whereas DQ909018-specific primers amplified products of ∼500 nt in length. Cloning and sequencing the latter products yielded a single sequence (not shown); using this information for 5′- and 3′-RACE approaches, we cloned and identified a coding sequence (CDS) of 981 bp (AY821800). Sequence analysis showed identity to cysteine proteases of other trematodes, including *C. sinensis* (85%) and *Paragonimus westermani* (55%) (Figure 1). The ORF encoded a zymogen with a deduced size of 326 amino acid residues (molecular mass 37,325 Daltons, theoretical pI, 5.45) including a predicted signal peptide of 18 residues in length and a prosegment of 95 residues that encoded a cathepsin F protease. We termed the putative enzyme encoded by this ORF, *Ov*-CF-1, and the new gene *Ov-cf-1*. These dimensions were similar to the predicted size of the recently reported cathepsin F, CsCF-6, of the closely related fluke, *C. sinensis* [13]. The ERFNAQ motif characteristic of cathepsins F and W (similar to the ERFNIN motif of cathepsins L, S and O, etc.) was conserved in the prosegment of *Ov*-CF-1 (Figure 1). The catalytic triad residues were present as Cys26, His160 and Asn180, numbering from the predicted amino-terminal methionine, Met1, residue of the mature enzyme. Other motifs including six conserved Cys residues likely involved in disulfide bridges were present. The molecular mass of the predicted mature enzyme of 213 amino acids was 23,811.92 Daltons, with a theoretical pI of 6.42. The mature enzyme included a single, predicted glycosylation site, NGS, at residue 109.

BlastP analysis undertaken with the 213 residues of the mature *Ov*-CF-1 protease as the query matched to peptidase family C1A, superfamily C1. Best matches were cathepsin Fs from *C. sinensis* and *Paragonimus*. The next best match was to cathepsin F of zebrafish, *Danio rerio*, NP_001071036. A TBLASTN search at using the 95 amino acid residues of the prosegment yielded strong matches to the prosegment of cathepsin F-like enzymes from *C. sinensis* AF093243 [13] and to *Paragonimus westermani*, DQ016551 [25] and also to schistosome and mammalian cathepsin Fs. (The transcriptome of *O. viverrini* indicates the presence of other family C1 cysteine proteases including cathepsins B and L [26].)

### The *Ov-cf-1* Gene Is Organized into Seven Exons

**Figure 2:**
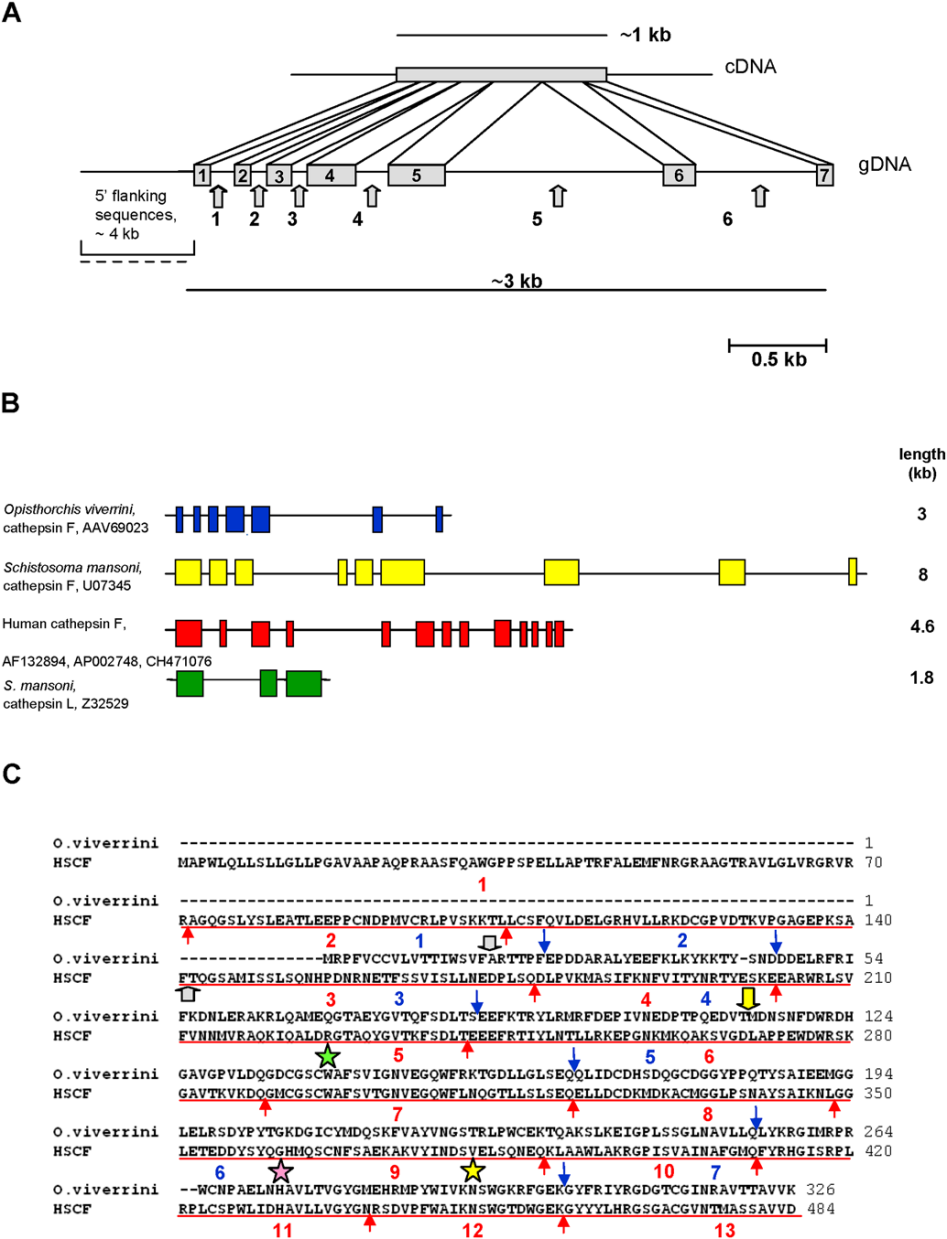
Genome structure of the cathepsin F gene of *Opisthorchis viverrini*. (A) Structure of the *O. viverrini* cathepsin F gene locus, as determined by nucleotide sequence analysis of a ∼3 kb PCR product of genomic DNA of *O. viverrini*. The top panel shows the size of the cDNA while the bottom panel shows a schematic of positions and relative sizes of the 7 exons and 6 introns (arrowed) that comprise the gene. The 5′-flanking region was generated by inverse PCR. (B) Schematic to compare the structure and exon numbers of the C1 family genes, including the cathepsin F genes of *O. viverrini*, *Schistosoma mansoni*, and *Homo sapiens*, and the cathepsin L gene of *S. mansoni*. Colored blocks represent exons, thin lines represent introns, and the asterisks identify the exon(s) that encodes the catalytic Cys residue. Database accession numbers are provided for the illustrated sequences. (C) Comparison of the exon/intron structures of *O. viverrini* cathepsin F and human cathepsin F. The two enzymes were aligned for maximal homology and the amino acid sequences corresponding to an exon for each protein was separated by red (human cathepsin F) or blue (*O. viverrini* cathepsin F) arrows. Exon numbering is shown below the blocks of amino acid sequences. Positions of the active site triad of Cys, His and Asn residues are indicated with stars. The position of signal sequence cleavage site is indicated with gray arrows, and the position of FhCL1 *trans*-processing cleavage site between prosegment and mature enzyme indicated with the yellow arrow.

A contiguous sequence of 7,368 bp composed of 5′-UTR and the genomic sequence of *O. viverrini* cysteine protease was assembled from iPCR and long range PCR fragments (GenBank FJ346536) (Figure S1). The contig extended 4,211 bp upstream of the predicted translation start site (ATG) of the gene. The entire genomic sequence of *O. viverrini* cysteine protease characterized from start codon referred to mRNA sequence to the end was 3,157 bp. By comparing the genomic and cDNA sequences of *Ov-cf-1* and by prediction of the splice sites, a gene structure of seven exons interrupted by six introns was determined for the genomic organization of the *Ov-cf-1* gene (Figure 2A). The sizes of exons 1 to 7 were >69, 69, 117, 240, 267, 145 and 113 bp, respectively (Table S1 and Figure 2A–2C). The length of introns 1 to 6 were 132, 43, 46, 192, 1,060 and 664 bp, respectively. The structure of cathepsin F-like genes from several species, cathepsin F from *S. mansoni*, human cathepsin F, and cathepsin L2 from *S. mansoni*, was compared with that of the *Ov-cf-1* gene (Figure 2B) since these three other genes were likely to be informative in terms of determination of orthology. *O*v*-cf-1* has 7 exons while *S. mansoni* CF1 and human cathepsin F have nine and 13 exons, respectively. As illustrated in Figure 2C, the six exon boundaries are conserved exactly or closely when compared with the exon/intron boundaries in human cathepsin F. Human cathepsin F is substantially longer than *O. viverrini* cathepsin F, 457 aa compared with 326 aa. The catalytic triad Cys, His, Asn residues were located in exons 4, 6 and 6, respectively; in comparison, the catalytic triad C, Cys, His, Asn residues of human cathepsin F are located on exons 6, 11 and 12. Exons 1, 2 and 3 of *Ov-cf-1* appear to be orthologous to exons 3, 4 and 5 of human cathepsin F. Further downstream, the human gene is interrupted by more introns than the *O. viverrini* gene yet the orthology remains apparent. Thus, exons 6–7, 8–10, 11–12, and 13 of human cathepsin F likely are the orthologues of exons 4, 5 and 6, respectively, of *Ov-cf-1*. As mentioned, the catalytic Cys, His and Asn residues are encoded by exons 7, 11 and 12 in the human cathepsin F gene, and exons 4, 6 and 6 in the *Opisthorchis* gene, further supporting the orthologous nature of the two genes.

The splice donor and acceptor sites of all six introns in the *Ov-cf-1* gene confirmed to the established consensus GT at the 5′-end of the intron and AG at the 3′ end [27] (Table S2). Blastn and tblastx searches were undertaken using the ∼4,200 bp upstream of the start codon, ATG. Several matches were obtained, specifically for query residues 1–250 which gave tblastx matches to a genomic sequence of the nurse shark *Ginglymostoma cirratum* (AC165195) (tblastx score, 106; E value, 4e-21) and the reverse transcriptase of a *Penelope*-like retrotransposon from *Schistosoma mansoni* (BK000685) (score, 100; E value, 2e-19) (Table S1). Similarity searches using tblastx of the intron sequences of *ov-cf-1* revealed several matches, including a match of intron five to the gene encoding PHGPx of *Clonorchis sinensis* and a match for intron six to a trinucleotide repeat containing 6a gene of *Mus musculus*.

### Phylogenetic Analysis of *O. viverrini* Cysteine Protease

**Figure 3:**
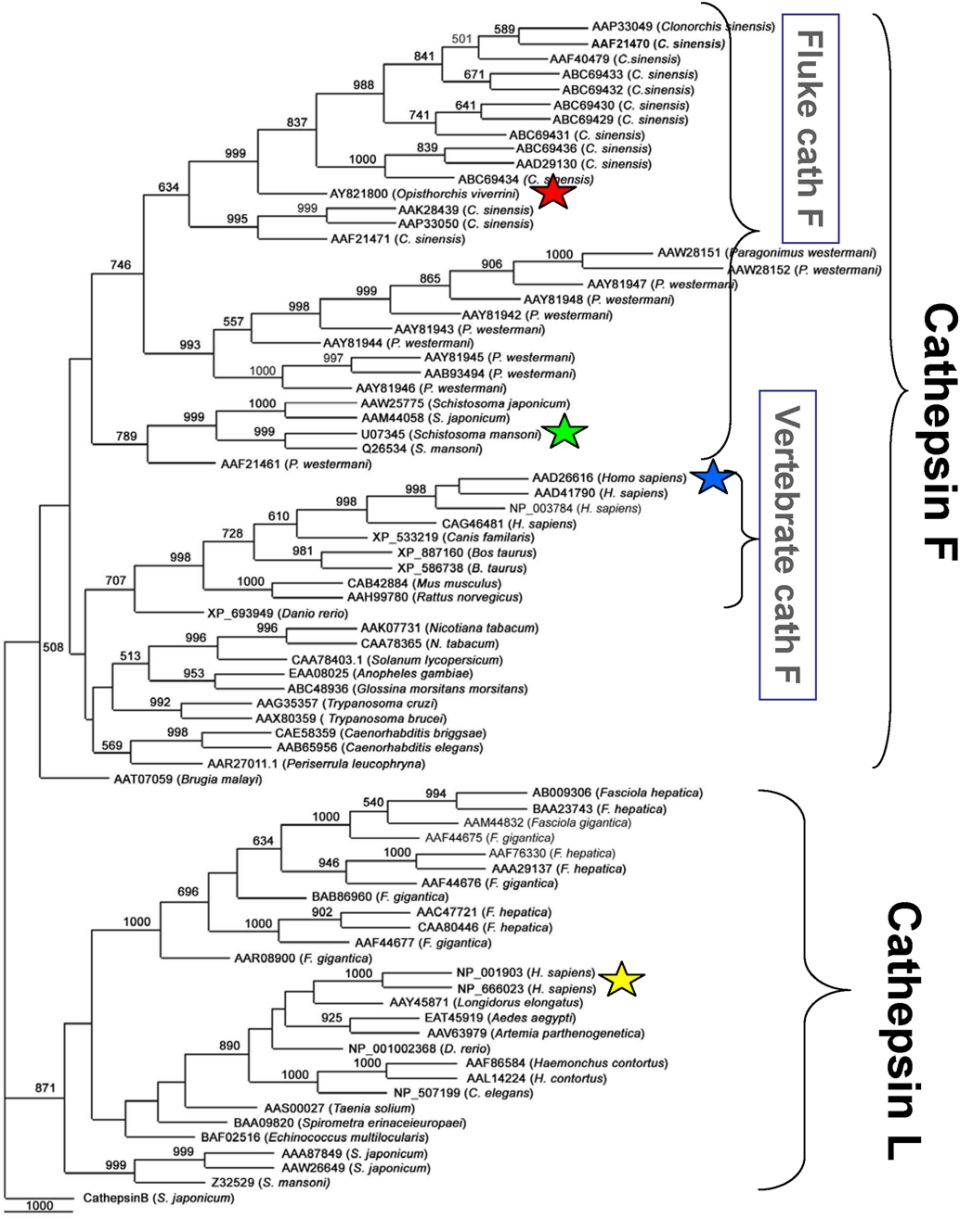
Neighbor joining tree. The tree revealed the phylogenetic relationship between the cathepsin F cysteine protease of *Opisthorchis viverrini* and homologous enzymes from ∼75 other informative species. Species names and GenBank accessions are provided on the branches. Bootstrap values of 1,000 replicates are provided at the nodes of the branches (bootstrap values less than 500 were omitted).

Phylogenetic analysis using >75 related sequences (primarily cathepsin F and cathepsin L like enzymes) confirmed the relationship of *Ov*-CF-1 with other cathepsin F proteases (Figure 3). *Ov*-CF-1 clustered with more than ten *C. sinensis* cysteine protease mRNA sequences. It also clustered with five or more cathepsin F-like sequences from the lung fluke, *P. westermani*, although the *Paragonimus* orthologues branched separately from the *Opisthorchis* and *Clonorchis* cathepsins F. Further, it clustered with the cathepsins F of the schistosomes *S. japonicum* and *S. mansoni*, although the schistosome orthologues branched separately from both the *Opisthorchis*/*Clonorchis* and the *Paragonimus* cathepsins F. Together the branches that included these fluke cathepsins F branched separately from mammalian cathepsins F, including human cathepsin F. The branch that included the mammalian cathepsins F clustered with another branch of cathepsin F-like enzymes from a diverse group of eukaryotes including potato, mosquito, *C. elegans* and trypanosomes. An unusual cathepsin F-like enzyme from the nematode *Brugia malayi* (AAT07059) [28] formed a basal branch of the cathepsin F-like sequences. Cathepsin L like sequences from *Fasciola* species, schistosomes, tapeworms, *Haemonchus contortus*, *Aedes aegypti*, human, and other species formed a completely distinct clade to the cathepsins F. The entire tree was rooted with the cathepsin B of *S. japonicum*.

### *Ov*-CF-1 Expressed in Gut of Fluke and Released into Liver of Infected Hamster

**Figure 4:**
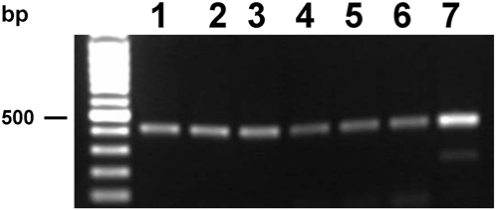
Transcription of *Opisthorchis viverrini* cysteine protease mRNA revealed by RT-PCR. Developmental stages examined: (lane 1), metacercariae (2), juvenile 1 week (3), juvenile 2 weeks (4), juvenile 3 weeks (5), adult worms (6) and *O. viverrini* cDNA library (7).

RT-PCR revealed that the *Ov-cf-1* mRNA was transcribed in eggs, metacercariae, immature and sexually mature forms of *O. viverrini*, including one-, two- and three-week-old juveniles and adult worms (Figure 4). Anti-*Ov*-CF-1 serum was employed to investigate the presence of the cathepsin F in ES products and the organ- and tissue-specific expression of the cysteine protease in sexually mature forms of the *O. viverrini* liver fluke. Immunoblot analysis of thiol-sepharose enriched cysteine protease activities indicated that *Ov*-CF-1 was secreted or excreted by adult *O. viverrini* (Figure S2). A reactive band at ∼30 kDa present in fractions of both ES and somatic fluke extracts. In addition, there were minor bands of reactivity at ∼37 kDa and 23 kDa in the somatic antigen preparation, and 23 kDa in the ES. Given that there is an *N*-linked glycosylation site in the mature enzyme, the immunoblot findings indicate that the mature enzyme is glycosylated and migrated at ∼30 kDa. We interpret the additionally bands as the unprocessed zymogen (∼37 kDa) and a non-glycosylated form of the mature enzyme (∼23 kDa). Together, the immunoblot findings indicated that the antiserum specifically recognized *Ov*-CF1.

**Figure 5:**
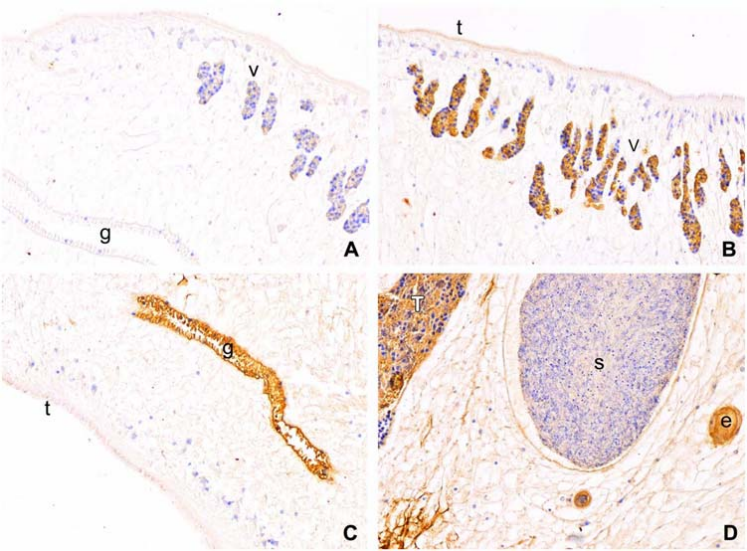
Immunolocalization of cathepsin F cysteine protease in adult *Opisthorchis viverrini* using thin sections of paraffin embedded worms probed with rabbit antiserum. (A) Representative section spanning the gut, vitellaria, parenchyma and tegument, probed with pre-immunization serum (negative control). Sections of adults, probed with the rabbit anti-cathepsin F serum, the vicinity of the tegument and vitellaria (B), tegument and gut (C), and testis, seminal receptacle, parenchyma and eggs (D). Gut (g), vitelline glands (v), egg (e) and testis (T) all showed strong positive reactions whereas the sperm seminal receptacle (s) was negative. The tegument (t) was faintly positive but the tegumental cells were negative for the cathepsin F cysteine protease. Immunoperoxidase staining, original magnification, ×100.

**Figure 6:**
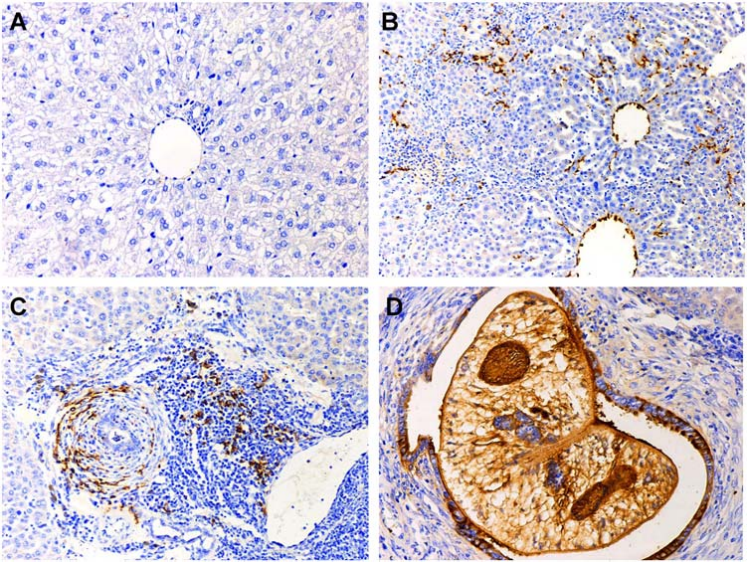
Immunolocalization of cathepsin F cysteine protease (*Ov*-CF-1) in *Opisthorchis viverrini* infected hamster liver. Thin sections of paraffin embedded liver tissues were probed with rabbit antiserum. (A) Representative section of liver from an uninfected hamster, spanning a portal triad including a secondary bile duct, probed with rabbit anti-*Ov*-CF-1serum (negative control). Infected hamster liver in the vicinity of the secondary bile ducts too small in internal diameter to include an adult fluke, probed with the rabbit anti-*Ov*-CF-1 serum (B and C). Immunoperoxidase stain (brown) indicates the presence of *Ov*-CF-1 in bile ducts epithelial cells (B) and in sinusoidal Kupffer and mononuclear cells (C). Section through bile duct containing an adult *O. viverrini*, showing strong reactivity to organs and tissues of the fluke (including the gut), and to the epithelial cells lining the infected bile duct (panel D). Immunoperoxidase staining, original magnification, ×100.

Immunohistochemical localization of *Ov*-CF-1 revealed strong expression in the gut, vitellaria, egg and testis (Figure 5B–5D). By contrast, control sections of flukes probed with pre-immunization serum showed no or little reactivity (Figure 5A). The intense immunolocalization to the luminal margin of the gut (Figure 5C) indicated strongly that the cathepsin F participates in proteolysis of ingested host tissues. In addition, sections through hamster bile ducts containing adult *O. viverrini* flukes confirmed the localization of *Ov*-CF-1 in organs and tissues of the fluke, including the gut and, notably, revealed the presence of *Ov*-CF-1 in epithelial cells lining the infected bile duct (Figure 6D). *Ov*-CF-1 also had accumulated in secondary bile ducts too small in internal diameter to include an adult fluke (Figure 6B) and in Kupffer cells and mononuclear cells lining sinuses of the liver (Figure 6C). Control sections from the same infected hamster probed with pre-immunization serum showed no or little reactivity (Figure 6A).

### Activation, Processing, and Biochemical Analysis of *Ov*-CF-1

**Figure 7:**
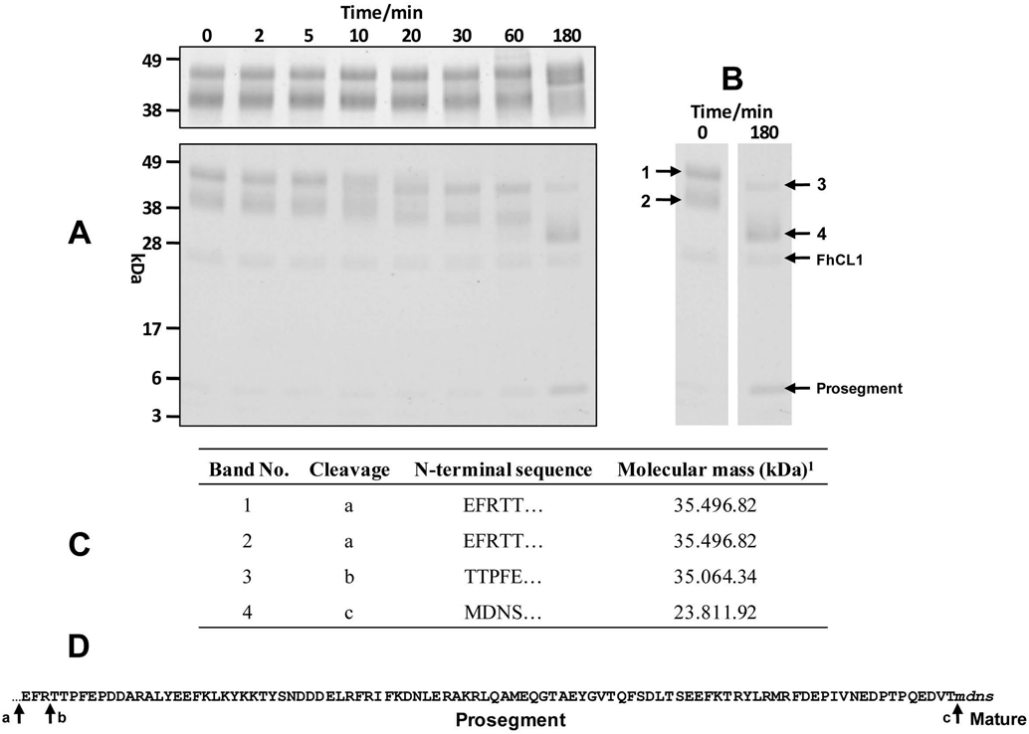
*Trans*-processing of *Ov*-CF-1 by *Fasciola hepatica* cathepsin L1 (FhCL1). (A) (Top) Purified recombinant *Ov*-CF-1 was incubated at pH 4.5 for 180 min. Aliquots of the reaction mixtures were removed at time 0, 2, 5, 10, 20, 30, 60 and 180 min and analyzed on 4–12% Bis-Tris NuPage gels (Invitrogen). Recombinant *Ov*-CF-1 is not capable of auto-activation at pH 4.5. The two bands shown are likely due to differential glycosylation as the recombinant was produced in yeast; (bottom) Purified recombinant *Ov*-CF-1 (50 µg) was incubated with fully activated mature FhCL1 (5 µg) for up to 180 min in 0.1 M sodium acetate, pH 4.5 (reaction volume 150 µl). Aliquots (15 µl) were removed from the mixtures at time 0, 2, 5, 10, 20, 30, 60 and 180 min and analyzed by 4–12% Bis-Tris NuPage gels. By 180 min marked *trans*-processing of *Ov*-CF-1 by FhCL1 had occurred. (B) Profiles of the *Ov*-CF-1 (time 0 min) and at 180 min of *trans*-processing with FhCL1 time showing the peptide bands (1–4) that were analyzed by N-terminal sequencing. The position of exogenously added FhCL1 and the released *Ov*-CF-1 prosegment are also shown. (C) N-terminal sequences obtained for each of the *Ov*-CF-1 peptides before and after *trans*-processing by FhCL1. (D) The cleavage sites identified by N-terminal sequences were also mapped onto the primary amino acid sequence of the *Ov*-CF-1 prosegment. The EF found at the N-terminal was introduced by the *Eco*R I cloning site used in the pPicZα expression vector.

The recombinant *Ov*-CF-1 purified from yeast culture medium resolved as two major protein bands migrating at 41 kDa and 47 kDa on 4–12% Bis-Tris NuPage gels (Figure 7A). Given the theoretical molecular mass of the recombinant *Ov*-CF-1 zymogen (including the *c-myc* and His_6_ tags) of ∼37.5 kDa, and given that both the 41 kDa and 47 kDa peptides share identical N-termini (EFRTT; Figure 7C), it is likely that the yeast-expressed enzyme exhibits differential addition of N-linked glycans. This is consistent with the smearing observed around both protein bands (*Ov*-CF-1 contains a single predicted *N*-linked glycosylation site, NGS, at residue 109; Figure 1). The yeast-expressed *Ov*-CF-1 zymogen displayed modest activity against the protein substrates hemoglobin and gelatin at acidic pH (4.5–6.0) (not shown). Assays undertaken with the fluorogenic substrate Z-Phe-Arg-NHMec revealed that the enzyme was active over a broad pH range, 4.5–8.0, with optimal activity at pH 5.5 (not shown). However, the apparent activity of the zymogen was very low, which is not surprising since the gel analysis did not reveal evidence of a fully processed and activated enzyme (Figure 7A).

**Figure 8:**
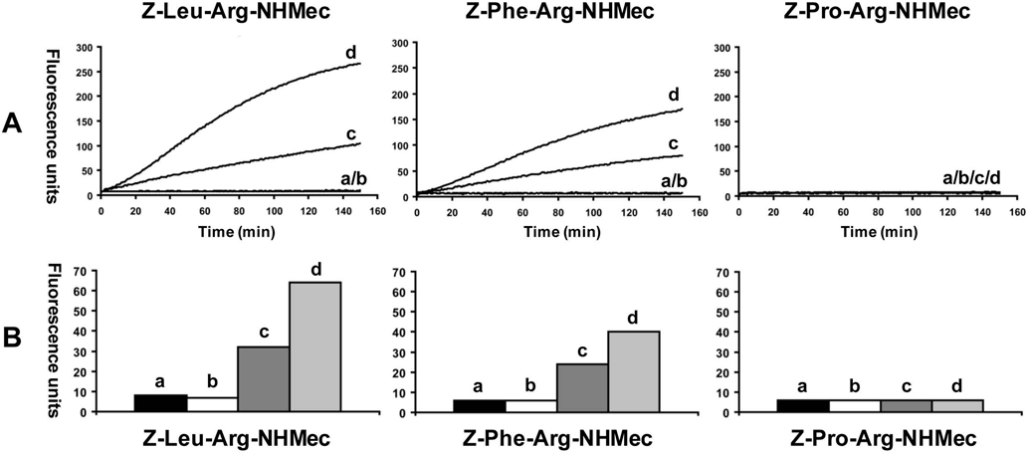
Exogenous activation of *Ov*-CF-1 by FhCL1 in the presence of peptidyl-NHMec substrates. (A) Initial rates of hydrolysis of the fluorogenic dipeptide substrates Z-Leu-Arg-NHMec, Z-Phe-Arg-NHMec and Z-Pro-Arg-NHMec measured by monitoring the release of the fluorogenic leaving group (−NHMec) over 150 min at 37°C. (B) Comparison of the hydrolysis of each fluorogenic peptide substrate within the linear range of the reactions (0–30 min) by recombinant *Ov*-CF-1 and FhCL1; relative fluorescence units are presented. The reactions included negative control (a); recombinant *Ov*-CF-1 alone (b); recombinant FhCL1 alone (c); *Ov*-CF-1 +FhCL1 (d).

Both cathepsin L and B cysteine proteases of trematodes such as *Schistosoma mansoni* and *Fasciola hepatica* have been shown to auto-catalytically remove the prosegment at low pH to release the fully active mature enzyme (see [29]). In order to investigate whether *Ov*-CF-1 was likewise capable of auto-catalytic processing, recombinant *Ov*-CF-1 was incubated for 180 min at pH 4.5, after which the reaction products were analyzed in Coomassie blue-stained gels. Evidence of auto-catalysis of the zymogen was not apparent (Figure 7A, upper panel). When similar reactions were performed in the presence of fluorogenic peptide substrates, no increase in enzyme activity of *Ov*-CF-1 was detected (see below, also Figure 8A). These data suggested that the recombinant *Ov*-CF-1 did not auto-catalytically process and activate. Subsequently, we investigated whether *Ov*-CF-1 could be *trans*-processed and activated by a functionally active *O. viverrini* asparaginyl endopeptidase [17] or a fully-activated recombinant cathepsin L cysteine protease from *F. hepatica* (termed FhCL1). *Ov*-CF-1 was incubated with asparaginyl endopeptidase or FhCL1 at a ratio of 10∶1, at pH 4.5, 37° C, and the reaction products monitored over 180 min by SDS-PAGE. No *trans*-processing was obvious with the asparaginyl endopeptidase (not shown); however, in the presence of FhCL1, the 41 kDa and 47 kDa species of *Ov*-CF-1 were clipped to progressively faster-migrating bands (Figure 7A, lower panel). By 180 min, a prominent band had accumulated at 30 kDa, together with a minor band of 43 kDa and a small peptide migrating at 5 kDa (Figure 7B). N-terminal sequencing confirmed that the 30 kDa band represented a fully mature enzyme generated by cleavage at Val-Thr ↓ Met (Figure 7C and 7D). Peptide sequencing established that the 43 kDa protein was produced by removal of just three residues (EFR) from the N-terminus of the 47 kDa *Ov*-CF-1 zymogen (Figure 7C and 7D). N-terminal sequencing of the 5 kDa band was inconclusive, but this peptide likely represented a remnant product of the liberated prosegment of *Ov*-CF-1 (Figure 7B).

Figure 8A presents the *trans*-processing of *Ov*-CF-1 by FhCL1 in real-time. In these studies, the *Ov*-CF-1 zymogen displayed very low activity (5–10 relative fluorescent units against both peptide substrates Z-Leu-Arg-NHMec and Z-Phe-Arg-NHMec). This activity did not significantly increase when the zymogen was incubated alone for 150 min at pH 4.5. However, following addition of FhCL1 to *Ov*-CF-1 the activity of the cathepsin F increased over the course of the experiment (Figure 8), demonstrating that exogenous *trans*-processing of the *Ov*-CF-1 prosegment generated a highly active, mature enzyme. By subtracting the activity of the FhCL1 when assayed alone against the substrates from that observed for the mixture of *Ov*-CF-1 and FhCL1, we estimated that mature *Ov*-CF-1 was >50 times more active (after 150 min) than its zymogen. From the exogenous activation in the presence of three fluorogenic peptides, Z-Leu-Arg-NHMec, Z-Phe-Arg-NHMec and Z-Pro-Arg-NHMec, we obtained information on the substrate specificity of *Ov*-CF-1. *Ov*-CF-1 had a similar specificity for residues in the P2 substrate as FhCL1; both enzymes accommodated the hydrophobic amino acids Phe and Leu but were incapable of cleaving peptides with the bulky residue Pro in the P2 position (Figure 8A and 8B). For FhCL1, this is consistent with the findings of Stack and co-workers [30]. Initial rates of hydrolysis of the substrates during the linear phase (0–30 min) revealed an affinity by *Ov*-CF-1 for the substrates in the order Z-Leu-Arg-NHMec >Z-Phe-Arg-NHMec (Figure 8B).

## Discussion

Cysteine proteases have been characterized in numerous infectious pathogens (e.g., [31]–[33]). In helminth parasites, their functions include excystation, tissue invasion, catabolism of host proteins for nutrition, and immunoevasion [34],[35]. Given the importance of *O. viverrini* in the etiology of CCA [1], [2], [4]–[6], there is impetus to characterize parasite antigens, including proteases, involved in the host-parasite relationship and pathophysiology of this food-borne fluke. Previously, we have determined the presence of abundant cysteine protease activity using the diagnostic peptide Z-Phe-Arg-NHMec in the ES products of adult *O. viverrini* and in other developmental stages [14] and shown that the transcriptome encodes numerous proteases [26]. More specifically, these findings demonstrated that *O. viverrini* expresses clan CA peptidases and suggested that abundant cysteine protease activity was present in metacercariae where it might be involved in cyst excystation during mammalian infection [14].

Cysteine proteases have been characterized in numerous infectious pathogens (e.g., [31]–[33]). In helminth parasites, their functions include excystation, tissue invasion, catabolism of host proteins for nutrition, and immunoevasion [34],[35]. Given the importance of *O. viverrini* in the etiology of CCA [1], [2], [4]–[6], there is impetus to characterize parasite antigens, including proteases, involved in the host-parasite relationship and pathophysiology of this food-borne fluke. Previously, we have determined the presence of abundant cysteine protease activity using the diagnostic peptide Z-Phe-Arg-NHMec in the ES products of adult *O. viverrini* and in other developmental stages [14] and shown that the transcriptome encodes numerous proteases [26]. More specifically, these findings demonstrated that *O. viverrini* expresses clan CA peptidases and suggested that abundant cysteine protease activity was present in metacercariae where it might be involved in cyst excystation during mammalian infection [14].

Here, we report that *O. viverrini* expresses a cathepsin F cysteine protease throughout its development and that the enzyme is released from the adult parasites which reside in the bile ducts. The cathepsin F may be responsible for the catalytic activity that we described previously and for which an EST was detected [14],[26]. Earlier studies have shown that both cathepsin L and cathepsin F cysteine proteases are abundant in several flukes, and that these two classes of proteases form distinct but closely related phylogenetic clades [36],[37]. Phylogenetic studies presented here show that the cathepsin F family of *O. viverrini* is most closely related the numerous cathepsin F-like transcripts expressed by another liver fluke, *C. sinensis*, which is informative since this parasite is also associated with cancer of the bile duct [38]. Whereas it is not yet clear whether *O. viverrini* also expresses and secretes cathepsin L, in addition to cathepsin F, transcripts that could encode cathepsin L are not abundant among the presently available *O. viverrini* ESTs, despite the fact that transcripts corresponding to several other Family C1 proteases, such as cathepsin B, are available [26]. *C. sinensis*, like *S. mansoni* [39],[40], *S. japonicum* [41] and *Paragonimus westermani* [25],[42],[43], appears to have both cathepsin F and cathepsin L -like enzymes [13]. In conspicuous contrast, *Fasciola hepatica* and *F. gigantica*, which also parasitize mammalian bile ducts, have a battery of cathepsin L enzymes yet no recorded cathepsin F [37],[44].

To date, four orthologous cathepsin F-like proteases of fluke parasites of humans have been functionally characterized. These are SmCF from *S. mansoni* [39],[40], Pw28CCP and related enzymes from *P. westermani* [25],[42],[43], CsCF-6 from *C. sinensis* [13] and *Ov*-CF-1 (this study). Cytochemical studies have shown that the cathepsin L and cathepsin F proteases of schistosomes localize to the gastrodermis of the gut and participate in extracellular digestion of ingested host tissues [40]. Caffrey and colleagues showed that promoter regions of the SmCF gene could drive reporter transgene expression in the gut of adult *S. mansoni* [45], thus providing strong support for a specific role for this protease in digestion of host blood [40]. CsCF-6 is also predominantly expressed in the gut of the adult *C. sinensis* where it is likely has a nutritional role [13]. In *F. hepatica*, cathepsin L1 and L2 are synthesized and secreted from gastrodermal epithelium and are essential for migration of the juvenile fluke through the intestinal wall and liver capsule, and for digestion of ingested blood and other host tissues [37],[44]. RNA interference of cathepsin L transcription blocks penetration of host tissues by the juvenile fluke [46]. Thus the localization of *Ov*-CF-1 in the gut of adult *O. viverrini* is consistent with findings in these related trematodes and consistent with a generalized function for both cathepsin F and L proteases in the degradation of host blood and other tissues.

The immunolocalization micrographs revealed that *Ov*-CF-1 was also expressed in the vitellaria, testis and eggs (Figure 5B and 5D). Transcripts of *Ov*-CF-1 were also detected in eggs (Figure 4). The role of the hydrolase in the reproductive organs of *Opisthorchis* has yet to be determined, particularly as reports on the immunolocalization of orthologues of cathepsin F in *C. sinensis* and *P. westermani* did not describe localization in reproductive structures [13],[43]. However, it has been reported that SmCL2, a cathepsin L of *S. mansoni*, is associated with the reproductive organs where it might activate phenol oxidase, an enzyme involved with eggshell formation, or with the seminal fluid [47]. *F. hepatica* cathepsin L has also been localized to the vitellaria [44]. It is possible that the cathepsin cysteine proteases in the reproductive structures of trematodes are encoded by different transcripts to those present in the gut but the protein products exhibit immunological cross-reactivity.

The immunolocalization investigations also revealed the presence of *Ov*-CF-1 in the biliary epithelium, and mononuclear cells and Kupffer cells in sinuses of the infected liver, indicating that release of *Ov*-CF-1 from the flukes into the bile and circulation (blood). It is possible that Kupffer cells and other macrophages phagocytose *Ov*-CF-1 and present the antigens to T cells, leading to inflammation. In this regard, *Ov*-CF-1 and other ES components (which are mitogenic in vitro; Sripa, unpublished) may stimulate inflammation and proliferation of biliary cells in the vicinity of the adult *O. viverrini* parasite. Such phenomena may promote cholangiocarcinogenesis.

Human cathepsin F and L are a papain-like cysteine proteases, with the MEROPS classification Clan CA, Family C1, Subfamily A (C01.018 and C01.032, respectively) [48]. Although the function(s) of cathepsin F has not been fully elucidated, it may play a regulatory role in processing the invariant chain that is associated with MHC class II [49],[50]. Cathepsin L has a wide tissue distribution and is found in lysosomes where its endopeptidase activity is a key catalyst of lysosomal proteolysis, although it also plays specific roles in generation of peptide antigens for the MHC II system and in spermatogenesis. It also has roles in pathological processes including tumor invasion, metastasis and arthritis [49]. Most residues of the prosegment of cathepsin L are conserved in cathepsin F, including the ERFNIN motif (ERFNAQ in cathepsins F and W) [51],[52]. A key structural difference between mammalian cathepsin F and other Family C1 proteases, including cathepsin L, is a cystatin domain within the long prosegment, the physiological role of which has not been established. It has been suggested that the mammalian cathepsin F gene evolved through a gene fusion between an ancestral cystatin and Clan CA cathepsin gene [53] (Figures 1 and 2). Whereas *Ov*-CF-1 and related fluke enzymes clearly display sequence identity to mature human cathepsin F, none so far reported include the cystatin domain within the prosegment. Therefore, if their genes represent the progeny of the ancestral cathepsin F gene, the hypothesized gene fusion of cathepsin F and cystatin genes must have occurred after the branching event in the tree of life that lead separately to trematodes (a Lophotrochozoan clade) and vertebrates (Deuterostomia).

In human cathepsin F, the two splice sites that interrupt exons 1–3 are conserved between cathepsin F and several cystatin genes [53], providing cogent evidence of a gene fusion during the evolution of human cathepsin F. The genome organization of human cathepsin F includes 13 exons separated by 12 introns; that of *Ov-cf-1* is simpler, with seven exons separated by six introns. Because of the absence of a cystatin domain from the fluke cathepsin, exons one and two of the human gene do not have orthologues in the *O. viverrini* gene. However, downstream of the cystatin sequence, orthology between the human and *O. viverrini* cathepsin F genes becomes unambiguous; the exon/intron boundaries are strongly conserved between the two genes (Figure 2C). Interestingly, the cathepsin F-like gene *Tci-cf-1* from the strongylid nematode *Teladorsagia circumcincta*, a parasite of the abomasum of sheep, does not encode a cystatin domain in its prosegment, although the prosegment of this nematode cathepsin F is ∼30 amino acids longer than the 95 residue prosegment of *Ov*-CF-1 and orthologues from other flukes [54]. Cathepsin F is secreted by the parasitic stages of *T. circumcincta*, including the L4 but not by the free-living larval stages, and is immunogenic. Given that some other nematodes including *Caenorhabditis elegans* and *Brugia malayi* do encode a cystatin domain in the prosegment of their cathepsin F orthologues [54], the evolutionary provenance of the cathepsin F/cystatin gene fusion remains obscure.

All cysteine proteases are synthesized as inactive zymogens which include a prosegment [55]. The prosegment lies across the active site, in reverse orientation to normal protein substrates, and prevents premature activation of the zymogen during trafficking and storage. Removal of the prosegment, mediated by protease clipping at the juncture between prosegment and mature enzyme, is essential to allow entry of macromolecular protein substrates into the active site cleft of the hydrolase. Dalton and colleagues proposed that prosegment removal occurs in helminth cathepsins by a two-step route of *trans*-processing to generate a small pool of activated enzymes which primes a rapid catalytic activation cascade [29]. The initial event might be performed by the same protease or a different protease, such as asparaginyl endopeptidase or cathepsin B or L. Our data showing that recombinant *Ov*-CF-1 did not undergo autocatalytic activation, despite displaying low-level activity, suggested that native *Ov*-CF-1 requires *trans*-processing. Stack and co-workers described how the junction between the prosegment and mature enzyme of human and helminth cysteine proteases consists of a non-conserved, randomly-structured motif that is susceptible to cleavage at several sites, depending on the processing enzyme [24]. Based on the three-dimensional structure of human cathepsin F [50] and multiple sequence alignments with helminth orthologues (Figure 1), we predict that the protease-susceptible region of *Ov*-CF-1 prosegment is the peptide PTPQEDVTMD. Since this does not include asparagine residues, it is not surprising that *Ov*-CF-1 was not *trans*-processed by the *O. viverrini* asparaginyl endopeptidase, even though this enzyme occurs in the gut of the adult [17]. Several gut-associated cathepsins of schistosomes and *Fasciola* do possess asparagine residues at the prosegment/mature enzyme juncture and, consequently, are *trans*-processed by asparaginyl endopeptidase (see [29]). On the other hand, here we have demonstrated *trans*-processing of *Ov*-CF-1 in vitro by the cathepsin L protease FhCL1 of *F. hepatica*. Cleavage of the prosegment occurred at PTPQEDVT↓MD, positioning a Val at P2, which is favored by FhCL1 [16],[56]. This cleavage resulted in a >50-fold increase in *Ov*-CF-1 activity. The ability of FhCL1 to *trans*-process *Ov*-CF-1 suggests that another endogenous Family C1 protease of *O. viverrini*, e.g. cathepsin B, processes and activates the cathepsin F within the adult gut.

Notwithstanding that cathepsins F and L belong to distinct phylogenetic clades [57] (Figure 3), our studies demonstrated that they share overlapping substrate preferences. Both *Ov*-CF-1 and FhCL1 accommodated substrates with hydrophobic residues in the P2 position; the P2 residue occupies the S2 subsite of the active site which primarily dictates the specificity of members of Clan CA. Additionally, both enzymes preferred Leu over Phe, whereas neither accommodated the bulky residue, proline. The selectively of FhCL1 for hydrophobic residues is likely the result of specific adaptation to the cleavage of mammalian hemoglobin, a substrate in which 42% of the component amino acids are Leu, Phe, Ala and Val [56]. Our limited substrate specificity analysis, however, has not yet revealed significant differences between the cathepsins F and L, which we suspect do exist. Indeed, we hypothesize that divergence of substrate preferences between cathepsins F and L is of central importance to the virulence and host species range of trematode parasites such as *O. viverrini*, *F. hepatica* and *S. mansoni*. Since phylogenetic divergence of the cathepsin F and L clades must have been driven by positive selection, by extension, elucidation of how this favors degradation of their respective target host macromolecules or tissues will inform our understanding of host-parasite adaptation.

Finally, given the extraordinary linkage between a metazoan parasite and a tumor [1],[4],[5],[38], characterization of the nature and action of secreted proteins of *O. viverrini* such as cathepsin F may provide insights into liver fluke induced cholangiocarcinogenesis, and indeed fundamental insights into carcinogenesis at large. In addition, cathepsin F may have potential as an intervention target including a vaccine candidate, given recent successes with chemotherapy targeting related enzymes in schistosomes [58] and with vaccines targeting cathepsin L of *Fasciola hepatica* [59]. Indeed, in view of the recent implementation of an acclaimed vaccination of adolescents against papilloma-virus infection to provide protection from cervical cancer [60], there is the tantalizing prospect that vaccination to prevent *O. viverrini* infection could provide protection against another infection-related cancer, liver fluke-induced cholangiocarcinoma.

## Supporting Information

Figure S1 Genomic DNA sequence of the gene locus encoding the cathepsin F of *Opisthorchis viverrini*. The sequence includes annotation to reveal the positions of the seven exons interrupted by six introns. The sequence has been assigned GenBank accession FJ346536.
(0.04 MB DOC)

Figure S2 Immunoblot analysis of 10% SDS-PAGE separated somatic (lane 1) and excretory secretory (ES) (lane 2) fractions (fractions eluted from thiol-sepharose, as described [14]) probed rabbit anti-*O. viverrini* cathepsin F serum (diluted 1∶1000). Anti rabbit IgG-conjugated to horse radish peroxidase (Invitrogen) was employed as the secondary antibody at a dilution of 1∶5000. After removal of the second antibody, signals were developed using a chemiluminescence detection system (Amersham), and captured on X-ray film (Kodak). On the left, positions of molecular size standards are indicated in black colored text. The positions of three reactive bands are indicated with the blue arrows, at 37, 30 and 23 kDa in the somatic antigen fraction (lane 1) and 30 and 23 kDa in the ES (lane 2). The mass of the major band, at 30 kDa, indicates it may be a glycosylated form of the mature enzyme of cathepsin F of *O. viverrini*.
(0.04 MB PDF)

Table S1 *Opisthorchis viverrini* cathepsin F-like cysteine protease gene locus, GenBank accession number, size and identities of exons, introns and flanking regions, as determined by tblastx searches of GenBank nr/nt collection of sequences
(0.05 MB DOC)

Table S2 Exon and intron boundaries, splice donor and splice acceptor sites in the gene encoding *Opisthorchis viverrini* cathepsin F cysteine protease, *Ov*-CF-1.
(0.04 MB DOC)
