# Supplementary material for: Cathepsin F Cysteine Protease of the Human Liver Fluke, Opisthorchis viverrini
Source: PLoS Negl Trop Dis. 2009 Mar 24;3(3):e398. doi: 10.1371/journal.pntd.0000398 (PMC2654340; doi:10.1371/journal.pntd.0000398)
Supplement: Figure S1 — Genomic DNA sequence of the gene locus encoding the cathepsin F of Opisthorchis viverrini. The sequence includes annotation to reveal the positions of the seven exons interrupted by six introns. The sequence has been assigned GenBank accession FJ346536. (0.04 MB DOC) [file pntd.0000398.s001.doc]

**Figure S1**. Genomic DNA sequence of the gene locus encoding the cathepsin F of *Opisthorchis viverrini*. The sequence includes annotation to reveal the positions of the seven exons interrupted by six introns. The sequence has been assigned GenBank accession FJ346536.

Gene locus - *Ov-cf-1*

Length 3,157 bp, start codon to stop codon inclusive; and 4,211 bp of upstream flanking sequence; total = a 7,368 bp contig of genomic DNA of *O. viverrini* at the gene locus, *Ov-cf-1*

**UPSTREAM FLANKING SEQUENCES:**

AAGCTTCAGTGCGGAAATTGTGACAAACACTACGTTGGACAAACAGGAAGGAAGCTCTCTACCAGGATAAATGAACACACACTTGCAACCAAAACGCACGGCCAATTTTCACTCGTTTTCGTACATGAGGATCAAGATGGACACAAACCCAACTGGAGGCCCGTTCACATATTGGCTCAAGCGAGAACAAAGAAAGAACGTGAATTCATAGAAGGATGGCATTCCATTGAAAAGCCACTGCAGAAAAAAAGAATTACGGAACTATGGAACGCAGAAGCGGACAAAATGGAAAGAAGCAAACAATCAGGCTGAAGCAGGGAAACTACCAAAAATAGAAGCCGCCGTCACAACCAACCAGATTCCACGCAACACAAACCGGGAACGGACCTGAAGTACCCCAAAGCAGCAATCCAGCAGACCAGTGGAATTAGAGGCATACAGACCACAGCACAACAAGAATCACGATCGGCCCAATAATAATACAACAGAAGGTATATGCCAACTAGCCAATCACATCACACGATATTAGATACTCTAATGCTCTAAACTACATAAGCGCAGCTAGGCACGGAGGATGCACCCAATCAAATACGCACTGACGATGTCTCCTAGACTGGGGACGAAACACTTGCAAGCCACTTGCCAAGCTCGGCGAACAGACCAATTGCCATCTGATCAACCAACAGTAAACGGAAACATAGGTAGCTCGGGTAGACAACTTGACAACTGCAATCTTCACACTGAATGCAGTCATCGATAATTTGGTCCTGAGCTGGCGACGTATTCCGCAAAAACCCATGTGTTTTCCCTAAGAAATGGTTGGTCCAGGAATCGCCTTTGGGTGGATTTGATTACTCACTGGTTTGACATGCATCAACAGTGCCAAGTAACGGTGCTGGTAATGCCGGTGGGGCATCAGTGGTTCGCGGAAGAGTCGACTGCGCTGCGTCATCTTCGCGTGCATTTTAACTTCGTACACTGGTTCTCGAGACTGCGTTTCAGGAAGTTAATTAGAGCGCGTGAGGGGTTGAGCCGCAGTGGTAAAAAAACTCATGCATTTCGATTCTCATTTGAATCATACACTGACTGTCACACCGAAATCTGCCGTATTCTTCTTCACCATGAAGAAATCCGACTTGTCTGTAACAGATGGATGTCGCCTGCAATGCTTAGAAAGCACCTGAATGTAACGGGAGAGGAAACTGCGTTACGGTCATCCTACGTGAAGAAAACAGTTCACAATTGCCTCTGCCCATGGCCATGTACACGAAAGTTGCGTAGAGAGGGGGTAGCGGAAATTCCGCTGGAAAGTGGCAGTTGCAATTTCACTGTACAGCATGAATGTTGTGGATTCCGTTCCGAACTGTCAAAACAGGTTCACACACCGAAGTCATTTGTCATCTCAGATGCTTTGAAGTGCAATCATGCAGCAGTTGCTCTATTTTTGCAATCCCCCGATTCCGAAAGACGTCCGTACACAACGGATACGATTTGATAATTTGGGCGAGTTTGACGCGCTGAGTGATCATTGCATAGCCATAAATGGTCCTACTTTGTGTGAAATTCCATAACTTCAGGTGTCTATCCAAACTCTCACCTTAAGAATTCGTGTAGGTGCCGGCAGATCTGAGTATTGGATCATATGGGAGGAATTCACGCGAAGTATCTTTGTCAGGTGCTCCAGTAGTGATAGAATGATCCTCAATGGAATCTGGAAAATTTTGTTCGATTTCTCTATCGGTTGAAACTGATGTCTTTGAATTTTTTTGTTGATATTTTTTTGAGATATTAGGCACGAAAGTGCACCTGCATGACAAAAAGATTACGCTATGTGATGAGTGTCAAAATCGAAACCAAAAGGCAAAGTAGCTGAGCGCCGAGAGTGACCGGGCTGACTTATGGAGATCCGTGGATTGGTCCCAATGATTTGTGGTCGAAACGCACGGTAACTTGCCGAACTCGCAGCATTTTCTTGGAATTCTTCACAACGAAAGCGAGAGGCAATATCTCTGTTTCTGTAAGATCCGGACGGTTAAGGATCTCACAGGACGATTGCGACTATTAGATTATACGCAGTTCGTAGCCAGTAGTCAATAGGGCAGTCAGACGGGGGCAGCAGACGATATATTGTAGACTGACAGGAACTGGCTCTACAGACAATTGTTATCCTATCACCAGCTTGGCATCCCTGTATGGTTACCATAATAAGGAGATGATGTCAACAGCGGTCGAATTATGTGAAAGTGTGTTTATTTCGACAGACTGATCAGCTGGTGTTTTACCGCGACGCATTTTATTTTTAGCCGAACGCCAATGTCCACTGAATGGAGTGTGACGTGTATCGGTGCCTTACTAAACGCGCCTGGTATTTGGTAGGCATTGTAATTGGTAATGTTTCAGGATCTCGATTAAAATCACTATGGTTCGGTGAAACGGCAGTCCTTCCATATAGTTGCGATCGTTAAATTAACCGTGAGATAGCTCGCAAGATGAATCCTACAAACGGAAGACAGACTCATCTGAAGCCCACAGAGAAATCTGGAGATCGACTTCTCCTGAGAACCGTTACACAATTGGACCCTCCTCATCCAATGCGTGGCTATTGGGTTTGTTAGTCACTTACCCTGCTTTTAACCCGAGAGCACGTCAGGCGAAAAACAGCTTAAACGAAATTTAACATGTGGCTAATGCGATTAGTTTACGTCCACGGAGTCCAAAGTTGACGGTGAGGCAAAGCTGCATCCCCCGGCGCGGAACACACATCGCCCGGTGCACGACAAGTGGGCTGTAATCCACCCTCGGTATAATTATGTGCGAAACATATATGACCTACGTCGCTGCATGCGTACATAATCTTCCCGTGACGGATTTGTTTGAGGAGACTAAATTCCGTAGCTGGAAGAACTACATCTCAAGGCATGATGGACTGGACTGTCACTTGGACCATCTGGACAATGATTTTACTCCGGTGAATAATGTTCGCGATTAAAATCGATATTTGAATCTTCATTTTGTGAGAGATGTACACTACCGAAGCGTCGAGGAAGCAAAGCAGATGAACGACTCCCCTCCAAAGAGCCCCTGGTGGTCACAGAAATCCAGCGAACCGGTTAATCCCTGCGTAATGGGAACAACCACGTTGGTTGGGCCTCACAGATAGTCCATCTCGGTGAGAGACCACGGTGTATAAGCAGGATGTATTGACCAACGTGGTTTTTTTCCAAGCACGTTCAGCTATTGTTTCCCATGTTGAAGGTGAAAGAGCACTTCAGTGATTGTAGGAAGATTCGTTACTGTTTAGAGCACTTTATCTCCAAATGGTTTCCCCTAGAAAATGCTTTCGTAGGAGTACTTACTGTGGTCTGATTGTGTATGTGAATGCACACTCTCACCGATAGTCAGCTGCAGACAGAAGTGAAGATTGGACGCCTGTATTATACGTACAGCAGATATGGATCCACACTTTATACAAGTAAACACGGTAAGTAAATGCTTTGGTCGTCTGACAGTCTATCAGATACAGAAACTAACTGGTAACGGAAAGTATTCACTCAGAAGGCGTCAAAATTCTCCGGTCATTAGGGGACGGCACTGGGAAGATTAATGCAGAGTAGTCAATGCTACTTGCGTCTGAAATTCCATGCAAACATGACACCTTACTAGAACTGTCCCGTAACTCCTTCTTGTTGGAAACGATAAATAGGCACGAAACATTTTCAAAACAATGGAAGTACGTTCCTATGTACCATATACGACTGTCAAGTTTTCGAAGTATTCTGTGACATTTTGGAGATCGTCGTTGAAAGTTCGGCCGGTTTTGAAGCATCAGTCCGGTTTGTTAAGTTGACTACGTTCTGCTGATGAACCCTGTCAGAGCAACGATTCGAACGTTTTTCGTTTGATAACCCTCCAGATAAAAAGTGACCCAATGTAGGAACGTGTAGTTTGAGCGTGAGTGGAGTTGTGCCTACGCAATCGGCTTTACAAGTGCACCTTCACGCGGTCCAAAGTTGAGTTCGAGGCGTGCTCCATCCTCCGGCGCAGAACACCTATCACATATTTTTCATATCTCATATTTTTCAAATCCGACTAAACCCATGGACTTTCGTCATGCTATCTCTGTGTGTTACGTAGACCTTTCACTTGAACTTTGATACAGAGTATGAGAATAAATCGGGAAGGAGAGAGCAGCAGGGTCCCATCACAACGAG

**EXON 1 (ORF only):**

**ATGCGACCTTTCGTGTGTTGCGTGTTGGTGACAACAATCTGGTCTGTCTTCGCCAGAACTACCCCATTC**

**INTRON 1:**

**GTGAGTTC**TTTCCCATTCATTTGTACATGGACAGTGCTTG**T**CCTCATATGCATGGCGCACTGTAAGCTAGCATCTGATTGTGCGATCTCATCAGTTGAGCAGTGAATGTTGAACATGTTTGATGAATTTCAG

**EXON 2:**

**GAGCCTGACGATGCCCGGGCACTATACGAGGAGTTCAAGCTGAAGTACAAGAAGACCTATTCAAATGAT**

**INTRON 2:**

**GTGCGCGC**ACATTTTTTTGTGACTCCGAACTCGACTGATTTAG

**EXON 3:**

**GATGATGAGCTTCGATTTAGAATTTTCAAAGACAACTTGGAGCGCGCCAAGAGACTGCAGGCAATGGAACAAGGGACAGCGGAGTATGGTGTAACGCAATTTTCCGACCTGACCAGT**

**INTRON 3:**

GTAAGCTGTTCTGACTGGAAGTGGAGTGGACTTGTTTTCTGTCTAG

**EXON 4:**

**GAGGAGTTCAAGACGCGGTATTTGAGGATGCGATTTGATGAGCCGATTGTCAATGAGGATCCCACCCCACAAGAAGATGTGACGATGGATAACAGCAATTTTGATTGGCGAGATCATGGTGCAGTCGGACCAGTATTGGACCAAGGAGATTGTGGTTCGTGCTGGGCATTTTCTGTGATTGGGAATGTCGAGGGTCAGTGGTTCCGTAAGACTGGGGATCTACTAGGTCTCAGTGAACAG**

**INTRON 4:**

***GTGAGCGA***ATTTCAGTGGGTATTTTCTGTTGAATCTGGTAGGTTGTGGTACCATGATTTTTTATAATGTTTCTACAACGCCATTTTGATTAAACGCTCCAGGTTCATTCACAGATCGTATGATTAGTGCGTCGTAATAACCGAATTTGCGTTGTGAAATAAGTGACCGTTGTGCTTTCATTTCTGTACCTAG

**EXON 5:**

**CAACTTATTGATTGCGACCATTCCGATCAGGGATGTGATGGCGGTTATCCCCCACAGACTTACAGTGCAATTGAAGAGATGGGCGGGTTGGAGCTCCGATCGGATTACCCGTACACAGGAAAGGACGGAATATGTTATATGGACCAATCGAAGTTCGTAGCCTATGTGAACGGTTCAACTCGCTTGCCATGGTGCGAAAAGACACAGGCGAAATCATTGAAGGAAATCGGTCCACTTTCTTCTGGTTTGAATGCCGTATTGCTCCAA**

**INTRON 5:**

***GTGAGTGA***TCTCGGTAGGACCTCTTGCTGTCAACTTTGTTACGTGTGATTTCTGGCAGGACAGCACTCACTATCGTCCGGAGAAGTAGAGTTGCCTTTACCGATGACACTTCGATGCTTTACTCCCTTTTCGCGTTACCAGGTTTTCGTTTTGTTTCGATTCCGATATCACACTTAGTTGTATATATATAACTCTATTTTATCCTATTGATTTGATATTAATGTTATGGTCTTTTGTATATTCTGAACCTCTGAACATTTCACAAAGTCTAACCCTCATATTTCTGTTATAACTTATTCTATGTTGATTCCTGTTATCCTGGTAACCAAAGCCAAAGAATCTCTATCGGATTGCCGCCACTGTTTCAGTTCACATACATACTCGGGGAGTTTCCGAAGGGTTTTTTTTCCTATGCTACTCCGCTCTTATCAACATCAAATATGCTATCTCCACCATCGTTCACCACACACTAATGCTGATTCTAGTAATGTTTTTAACCTGACGACCGTTTTATAGGGATTTTTATTCGGGTTTTGTTCCCTGGGTACTGTGAACGCATTCAAGTGCCATAGTAGTCGGGGGCGAGATGGCCCAAGTGTTTAGAGCGCGAATTCACCGACCGGAAGGTCCGTGGTTCCAACCCGACCTCTGCCACCCTACTTCCCCTGGTCCTGGCTTGGGCAACCTGACAGTATCCCAGCCCTCGTGCTTCCTCCGGGTGGCACGGTAGCTAGGTACCGAGAAAGTGTTACAGCTGAACGATTTTTTTAGTAATCGGAATAACCATCACGGATGGGTATCGGCAGTCGTGACTGATTGTTGCAATAAATGGATGGGATAGCGACGAAGTAAGTACATGCGGACCAGCTGAATGCCAATGAAACTTAAAATATGTTTCTTCTGTCTGGTTCCAATGCAGGATGGTAAATGCCCGGTCAAACTTGGAACGGCTGTCAGCCCACTTTATAGTTCAACAGGCCAAATATCTTATTATGCCTGGTGAAATTCAGATAACCCTTCCCCCATTATTCCGTTTTTCTTTTATTTCGATTGTCATCAG

**EXON 6:**

**CTGTACAAACGAGGAATCATGCGTCCCAGATGGTGCAATCCAGCTGAACTGAACCACGCTGTTCTCACAGTCGGCTACGGCATGGAGCATAGAATGCCTTACTGGATAGTGAAAAACAGTTGGGGCAAACGTTTTGGAGAAAAG**

**INTRON 6:**

***GTCAGTGG***CTTTTATTACAGAAGTGCGGTGGCACACTGGTTTCTCACCTACCATCAGTTTGGCACTCCGTTGTTTCTCCGGCAAATTTCAGAGTATACAACGATTCCACTAACAACATGGATACTACCAACGCAGTTATATTTCCAATCTATAAGATACATCCCCTTCGATTAGGACCAAACCTTTAAAATTCCTATGCCACTCGGTTTGTTTTACGGTTCGAATTCAAGTTTATCTACCGATCGGCTTTCACAGATAAAATGACACATTTTACCTGTATTTGCTCCAAGTCAGAATGAACACTGATCCCCTTCTTAGATCATAATTCCGGTGTCATTAGTAGTCAGTCGGGTTGAGTTTGGTAACTGACTTGCAAACGCCTCGCCATCATTCGAGATGTTACCAGTTCGCGGTCGGGTGAGTCTTTTCATTGGGTGTCTTCTCATTTCGGCTGAAAGACTAATCAGCGAAGTTTTAGAGAACCACTGGGAGTACTTTACTTGGTTGCCTCTCGTTGTCACTGCCCCGGAGGTCACTAAGTTGTCTTTGCTGTCTCAGTCGCGAACTGTGAATCTTGATGCAATCAACGTTGGCTGGTCACTAGTGAAATAAACACTGCTGTTCTGTCCAAAATGTCTCATGT***GAATCCCGCTTCTGTTTTCA***

**EXON 7:**

**GGCTATTTCCGTATCTACCGAGGAGATGGAACTTGCGGAATCAACCGCGCAGTTACTACAGCCGTTGTCAAA**TAGACCAACTCCATGTTGATATTTTGCCCACATAGACAATAA

**(3’UTR:** TAGACCAACTCCATGTTGATATTTTGCCCACATAGACAATAA)
