# Supplementary material for: Cathepsin F Cysteine Protease of the Human Liver Fluke, Opisthorchis viverrini
Source: PLoS Negl Trop Dis. 2009 Mar 24;3(3):e398. doi: 10.1371/journal.pntd.0000398 (PMC2654340; doi:10.1371/journal.pntd.0000398)

Figure S2. Immunoblot analysis of 10% SDS-PAGE separated somatic (lane 1) and excretory secretory (ES) (lane 2) fractions (fractions eluted from thiol-sepharose, as described [14]) probed rabbit anti-*O. viverrini* cathepsin F serum (diluted 1:1000). Anti rabbit IgG-conjugated to horse radish peroxidase (Invitrogen) was employed as the secondary antibody at a dilution of 1:5000. After removal of the second antibody, signals were developed using a chemiluminescence detection system (Amersham), and captured on X-ray film (Kodak). On the left, positions of molecular size standards are indicated in black colored text. The positions of three reactive bands are indicated with the blue arrows, at 37, 30 and 23 kDa in the somatic antigen fraction (lane 1) and 30 and 23 kDa in the ES (lane 2). The mass of the major band, at 30 kDa, indicates it may be a glycosylated form of the mature enzyme of cathepsin F of *O. viverrini*.

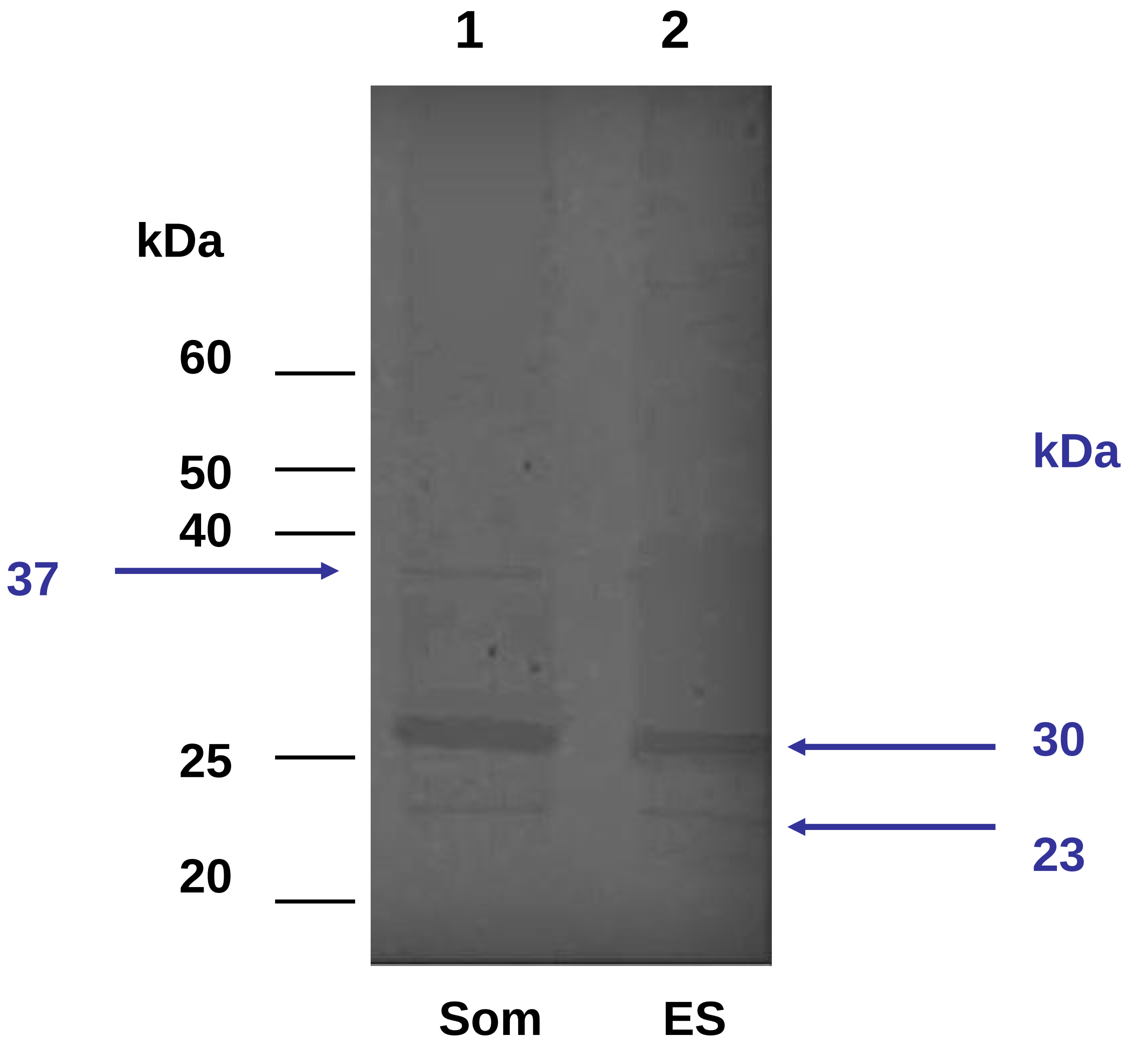

Supplement: Figure S2 — Immunoblot analysis of 10% SDS-PAGE separated somatic (lane 1) and excretory secretory (ES) (lane 2) fractions (fractions eluted from thiol-sepharose, as described [14]) probed rabbit anti-O. viverrini cathepsin F serum (diluted 1∶1000). Anti rabbit IgG-conjugated to horse radish peroxidase (Invitrogen) was employed as the secondary antibody at a dilution of 1∶5000. After removal of the second antibody, signals were developed using a chemiluminescence detection system (Amersham), and captured on X-ray film (Kodak). On the left, positions of molecular size standards are indicated in black colored text. The positions of three reactive bands are indicated with the blue arrows, at 37, 30 and 23 kDa in the somatic antigen fraction (lane 1) and 30 and 23 kDa in the ES (lane 2). The mass of the major band, at 30 kDa, indicates it may be a glycosylated form of the mature enzyme of cathepsin F of O. viverrini. (0.04 MB PDF) [file pntd.0000398.s002.pdf]
