# Supplementary material for: Cathepsin F Cysteine Protease of the Human Liver Fluke, Opisthorchis viverrini
Source: PLoS Negl Trop Dis. 2009 Mar 24;3(3):e398. doi: 10.1371/journal.pntd.0000398 (PMC2654340; doi:10.1371/journal.pntd.0000398)
Supplement: Table S1 — Opisthorchis viverrini cathepsin F-like cysteine protease gene locus, GenBank accession number, size and identities of exons, introns and flanking regions, as determined by tblastx searches of GenBank nr/nt collection of sequences (0.05 MB DOC) [file pntd.0000398.s003.doc]

**Table S1. *Opisthorchis viverrini* cathepsin F-like cysteine protease gene locus, GenBank accession number, size and identities of exons, introns and flanking regions, as determined by tblastx searches of GenBank nr/nt collection of sequences.**

| **Structure** | **Nucleotide position** | **Length (bp)** | **Identification** | **BLAST match to accession number (tblastx score [bits]; E value)** |
| --- | --- | --- | --- | --- |
| Upstream flank | 1-246 | 246 | Genome of nurse shark, *Ginglymostoma cirratum*  Reverse transcriptase of *Penelope* retrotransposon from *Schistosoma mansoni* | AC165195 (106; 4e-21)  BK000685 (100; 2e-19) |
| Upstream flank | 247-4211 | 3965 | Weak matches to *Drosophila melanogaster* chromosome X; *Paragonimus westermani* phospholipid hydroperoxide glutathione peroxidase isoform 2  AGAP001330-PA of *Anopheles gambiae*  *Branchiostoma floridae* hypothetical protein | AE014298 (42.3; 1.1)  DQ454160 (40.9; 2.9)  XM_321814 (33.6; 0.63)  XM_002212584 (40.9; 3.0) |
| Exon 1 | 4212-4280 | >69 | *Opisthorchis viverrini* cysteine protease mRNA  *Clonorchis sinensis* cathepsin F precursor | AY821800 (68, 5e-10)  DQ909018 (52, 2e-05) |
| Intron 1 | 4281-4412 | 132 | Weak match to *Bos taurus* X-inactivation center region | AJ421481 (38; 0.43) |
| Exon 2 | 4413-4481 | 69 | *Opisthorchis viverrini* cysteine protease mRNA  *Clonorchis sinensis* cathepsin F precursor | AY821800 (58, 7e-07)  DQ909018 (55, 3e-06) |
| Intron 2 | 4482-4524 | 43 | No significant similarity found |  |
| Exon 3 | 4525-4641 | 117 | *Opisthorchis viverrini* cysteine protease mRNA  *Clonorchis sinensis* cathepsin F precursor | AY821800 (97, 1e-18)  DQ909018 (83, 1e-14) |
| Intron 3 | 4642-4687 | 46 | No significant matches |  |
| Exon 4 | 4688-4927 | 240 | *O. viverrini* cysteine protease mRNA  *Clonorchis sinensis* cysteine protease (CP12) | AY821800 (207, 2e-51)  DQ346212 (186, 4e-45) |
| Intron 4 | 4928-5119 | 192 | *Dictyostelium discoideum* AX4 chromosome 5 DDB0232774.02, whole genome shotgun | NW_001263750 (37; 0.18) |
| Exon 5 | 5120-5386 | 267 | *O. viverrini* cysteine protease mRNA  *Clonorchis sinensis* cysteine protease (CP11) | AY821800 (222, 4e-56)  DQ346211 (173, 3e-41) |
| Intron 5 | 5387-6446 | 1060 | *Clonorchis sinensis* phospholipid hydroperoxide glutathione peroxidase isoform 2 | EF056482 (64; 4e-15) |
| Exon 6 | 6447-6591 | 145 | *Opisthorchis viverrini* cysteine protease mRNA,  *Clonorchis sinensis* cysteine proteinase 1 | AY821800 (8e-29)  AY273802 (4e-20) |
| Intron 6 | 6592-7255 | 663 | Weak match *to Mus musculus* trinucleotide repeat containing 6a | BC023872 (30; 8.30) |
| Exon 7 | 7256-7327 | 113 | *Opisthorchis viverrini* cysteine protease mRNA  *Clonorchis sinensis* cathepsin F precursor | AY821800 (65; 4e-09)  DQ909018 (56; 3e-06) |
| 3’UTR | 7328-7368 | 41 | *Opisthorchis viverrini* cysteine protease mRNA | AY82100 (38; 0.44) |
|  |  |  |  |  |
