# Supplementary material for: Cathepsin F Cysteine Protease of the Human Liver Fluke, Opisthorchis viverrini
Source: PLoS Negl Trop Dis. 2009 Mar 24;3(3):e398. doi: 10.1371/journal.pntd.0000398 (PMC2654340; doi:10.1371/journal.pntd.0000398)
Supplement: Table S2 — Exon and intron boundaries, splice donor and splice acceptor sites in the gene encoding Opisthorchis viverrini cathepsin F cysteine protease, Ov-CF-1. (0.04 MB DOC) [file pntd.0000398.s004.doc]

**Table S2**. Exon and intron boundaries, splice donor and splice acceptor sites in the gene encoding *Opisthorchis viverrini* cathepsin F cysteine protease, *Ov*-CF-1.

| **Exon** | **Length** | **Donor** |  | **Intron** | **Length** | **Acceptor** |  |
| --- | --- | --- | --- | --- | --- | --- | --- |
|  |  | **Exon** | **Intron** |  |  | **Intron** | **Exon** |
| 1 | >69 | CCATTC | gtgagttc | 1 | 132 | aatttcag | GAGCCT |
| 2 | 69 | AATGAT | gtgcgcgc | 2 | 43 | ctgatttag | GATGAT |
| 3 | 117 | ACCAGT | gtaagctg | 3 | 46 | tctgtctag | GAGGAG |
| 4 | 240 | GAACAG | gtgagcga | 4 | 192 | tgtacctag | CAACTT |
| 5 | 267 | CTCCAA | gtgagtga | 5 | 1060 | ttgtcatcag | CTGTAC |
| 6 | 145 | AAAAGG | gtcagtgg | 6 | 664 | tgttttcag | GCTATT |
| 7 | 114 |  |  |  |  |  |  |

.
